# Fossils reshape the Sternorrhyncha evolutionary tree (Insecta, Hemiptera)

**DOI:** 10.1038/s41598-020-68220-x

**Published:** 2020-07-09

**Authors:** Jowita Drohojowska, Jacek Szwedo, Dagmara Żyła, Di-Ying Huang, Patrick Müller

**Affiliations:** 1 Institute of Biology, Biotechnology and Environmental Protection, University of Silesia, 9, Bankowa St., 40-007 Katowice, Poland; 2 Laboratory of Evolutionary Entomology and Museum of Amber Inclusions, Department of Invertebrate Zoology and Parasitology, University of Gdańsk, 59, Wita Stwosza St., 80-308 Gdańsk, Poland; 3 Department of Ecology, Evolution and Organismal Biology, Iowa State University, Ames, IA USA; 4 State Key Laboratory of Palaeobiology and Stratigraphy, Center for Excellence in Life and Paleoenvironment, Nanjing Institute of Geology and Palaeontology, Chinese Academy of Sciences, Nanjing, 210008 China; 5 Kaeshofen, Germany; 6 Amber Study Group, c/o Geological-Palaeontological Museum of the University of Hamburg, Bundesstraße 55, 20146 Hamburg, Germany

**Keywords:** Entomology, Palaeoecology, Palaeontology, Taxonomy

## Abstract

The Sternorrhyncha, which comprise about 18,700 described recent species, is a suborder of the Hemiptera, one of big five most diverse insect orders. In the modern fauna, these tiny phytophages comprise insects of great ecological and economic importance, like aphids (Aphidomorpha), scale insects (Coccidomorpha), whiteflies (Aleyrodomorpha) and psyllids (Psylloidea). Their evolutionary history can be traced back to the Late Carboniferous, but the early stages of their evolution and diversification is poorly understood, with two known extinct groups—Pincombeomorpha and Naibiomorpha variously placed in classifications and relationships hypotheses. Most of the recent Sternorrhyncha groups radiated rapidly during the Cretaceous. Here we report the new finding of very specialised sternorrhynchans found as inclusions in mid-Cretaceous amber from Kachin state (northern Myanmar), which represent another extinct lineage within this hemipteran suborder. These fossils, proposed to be placed in a new infraorder, are revealed to be related to whiteflies and psyllids. We present, also for the first time, the results of phylogenetic analyses covering extinct and extant lineages of the Sternorrhyncha.

The Hemiptera is an ancient insect order, demonstrating extraordinary life histories and highly specialized morphological adaptations, as they have exploited diverse habitats and food sources through over 300 million years of their evolution. Hemiptera is one of the Big Five insect orders (with Coleoptera, Diptera, Lepidoptera and Hymenoptera), the most diversified and speciose orders among all insects, the largest non-holometabolous order of insects, representing approximately 7% of metazoan diversity. The Hemiptera currently contains around 320 extant and extinct families, which is the highest number among all insect orders^1^, with over 110,000 species already described^2–4^. The order Hemiptera is subdivided into six suborders^1^—extinct Paleorrhyncha (archescytinoids), Sternorrhyncha (modern aphids, scale insects, whiteflies, jumping plantlice, and their extinct relatives), Fulgoromorpha (planthoppers), Cicadomorpha (cicadas, froghoppers, leafhoppers, treehoppers, and number of extinct groups), Coleorrhyncha (moss bugs) and Heteroptera (true bugs).

Representatives of the Sternorrhyncha are tiny sucking phytophagous insects, representing nearly 19,000 described extant and extinct species distributed worldwide. They are highly diverse morphologically and ecologically, containing several extant infraorders Aphidomorpha, Coccidomorpha, Aleyrodomorpha) and Psyllodea, as well as extinct ones Naibiomorpha and Pincombeomorpha^1,4^. Both the fossil record from Moscovian of Avion^5^ and molecular divergence estimation^6^ show that the group was present during the Carboniferous. Sternorrhyncha have been evolving and diversifying for over 300 million years, but their fossils are less numerous than fossils of euhemipteran lineages (Fulgoromorpha, Cicadomorpha, Coleorrhyncha, and Heteroptera).

The consensus is that the Sternorrhyncha are a monophyletic lineage, but their internal classification is still an object of debate. Their sedentary lifestyles coupled with phloem-feeding behaviours in these insects, which behave as plant parasites, have driven morphological reductions and losses, neotenous females, extreme sexual dimorphism, and convergently derived morphological characters that would otherwise be useful in phylogenetic analyses. Thus, reconstructing the relationships of Sternorrhyncha is rather challenging. While Aphidomorpha and Coccidomorpha seem to be closely related, the placement of Naibiomorpha remains disputed. This group was placed within Aphidomorpha^8^ or in Coccidomorpha^9^. The Pincombeomorpha seems to form a distinct lineage together with Aphidiformes (i.e. Aphidomorpha + Naibiomorpha + Coccidomorpha). The second clade of Sternorrhycha—Psylliformes contains Aleyrodomorpha with Aleyrodidae and Psylloidea, the latter recently united with Protopsyllidioidea as Psyllodea^1^. Grimaldi^10^ stated that Protopsyllidiidae, which was once placed in Pincombeoidea^11^, should be placed as a sister group of all remaining Sternorrhyncha. However, in that analysis, representatives of the other extinct sternorrhynchan groups such as Pincombeomorpha and Naibioidea (Naibiomorpha) were not included. As a result of all these proposals, Psyllodea, as recently recognised^1^, seems to be a paraphyletic group and Protopsyllidiidae are not deemed to be direct ancestors of Psylloidea^12^. Recently, the morphological features, taxonomic content and classification of Protopsyllidioidea were reanalysed and a new hypothesis of their relationships was proposed, with Protopsyllidiidae as sister group to the Psylloidea + Aleyrodoidea clade^13^. Drohojowska^14^ postulated that Liadopsyllidae could be a sister group to the Psylloidea + Aleyrodoidea clade, based on morphological analysis of extant and extinct taxa.

The fossils described below are so morphologically remote and disparate from the other extinct and extant groups, that they cannot be placed in any of already proposed groups. They can be recognised as sternorrhynchan insect, based on characters of the head, thorax and wing venation. This motivated us to apply a phylogenetic approach to resolve the systematic position of the studied fossils.

## Results

### Phylogenetic analysis

We conducted Bayesian Inference (BI) and Maximum Parsimony (MP) analyses using morphological data to place the fossil taxa and resolve the relationships within Sternorrhyncha. Therefore, we mainly included those morphological characters that were also discernible in the fossils that were selected. The data matrix used for the analysis consisted of 10 taxa (Fulgoromorpha taken as an outgroup, and 9 Sternorrhyncha ingroups, including extinct groups, see Supplementary information 1 Table S1) and 42 characters (see Supplementary information 1 Table S2). The characters were treated as non-additive and unordered. The list of characters and the nexus file containing the character matrix is available in Appendix (Tables S1 and S2).

**Figure 1:**
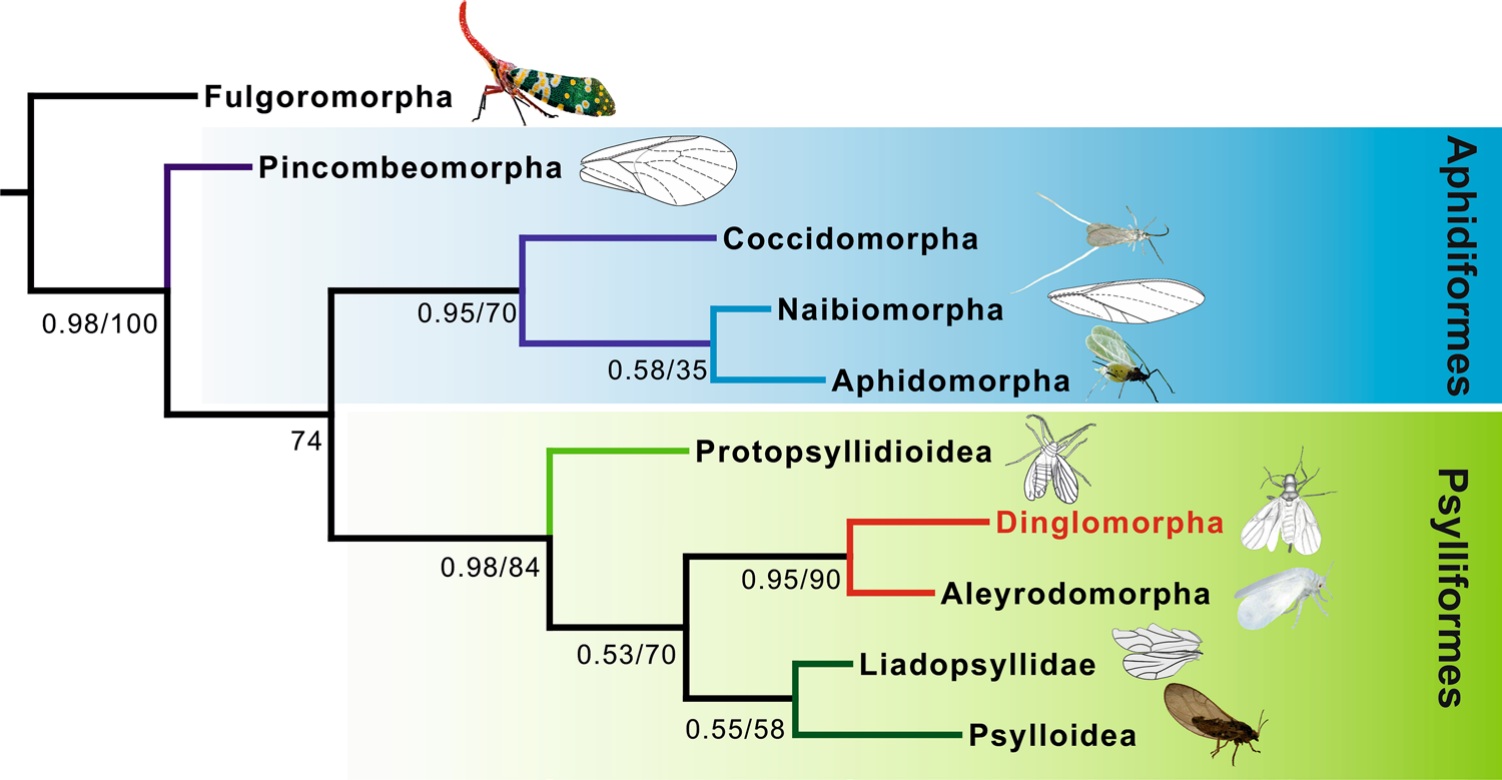
Phylogenetic position of *Dingla shagria* gen. sp. nov. on most parsimonius tree. Numbers at nodes represent posterior probabilities and bootstrap values. Image of planthopper *Pyrops candelaria*: Max Pixel Public Domain CC0 (modified); pincombeid *Pincombea* sp. redrawn from^46^; male scale insect: Pavel Kirillov CC-BY-SA2.0 (modified); *Coccavus supercubitus* redrawn from^46^; aphid *Macrosiphum rosae*: Karl 432 CC-BY-SA4.0 (modified); protopsyllidiid *Poljanka hirsuta* redrawn from^47^; liadopsyllid *Liadopsylla apedetica* redrawn from^48^; whitefly *Aleyrodes proletella*: Amada44 CC-BY-SA4.0 (modified); psyllid *Trioza urticae* photo by Jowita Drohojowska.

The detailed results of phylogenetic analyses are presented in the Appendix. Both phylogenetic methods (MP and BI) were highly congruent in their resultant topologies (Supplementary information 1 Figs S1, S2a–c). According to the resulting phylogenies, the fossil described below forms a group of its own (Fig. 1), included in a clade of Psylliformes, related to Psyllodea and Aleyrodomorpha, but deserving of recognition as a different infraorder.

### Systematic palaeontology

Order Hemiptera Linnaeus, 1758.

Suborder Sternorrhyncha Amyot et Audinet-Serville, 1843.

Clade Psylliformes sensu Schlee, 1969.

### Dinglomorpha Szwedo & Drohojowska infraord. nov

#### Diagnosis

Fore wing with costal veins complex carinate (Pc carinate as in Psylliformes), ScP present as separate fold at base of common stem R + MP + CuA (unique character); common stem R + MP + CuA weakened at base (unique character); areola postica reduced (homoplasy with Aleyrodoidea); clavus present, with single claval vein A_1_. Hypandrium present as small plate (as in Psylliformes).

### Dingloidea Szwedo & Drohojowska superfam. nov

#### Diagnosis

Fore wing membranous with modified venation—veins thickened, areola postica reduced; antennae 10-segmented; 3 ocelli present; stem MP present, connected with RP and CuA; abdomen widely fused with thorax; no wax glands on sternites.

### Dinglidae Szwedo & Drohojowska fam. nov

urn:lsid:zoobank.org:act:D0A1C785-62D3-4E07-9A3B-FFAE3C13B704.

#### Type genus Dingla

Szwedo et Drohojowska **gen. nov.**; by present designation.

#### Diagnosis

Imago. Head with compound eyes narrower than thorax. Eyes entirely rounded, postocular tumosity present; lateral ocelli placed dorsolaterally, near anterior angle of compound eye in dorsal view, median ocellus present. Antennae 10-segmented, with bases in frons to compound eyes, rhinaria scarce (?). Pronotum in mid line longer than mesopraescutum. Fore wing with thickened costal margin, basal portion of stem R + MP + CuA weak, distal portion of stem R + MP + CuA convex, forked at about half of fore wing length, branch RA short; pterostigmal area thickened. Common stem MP + CuA short, branches RP, MP and CuA parallel on membrane. Rostrum reaching metacoxae. Metacoxa without meracanthus. Metadistitarsomere longer than metabasitarsomere, claws distinct, long and narrow, no distinct additional tarsal structures. Male anal tube long. Hypandrium in form of small plate, styli long, narrow and acutely hooked at apex.

### *Dingla* Szwedo & Drohojowska gen. nov

LSID urn:lsid:zoobank.org:act:5053D386-4A13-445C-8036-9C69D885561F.

#### Type species Dingla shagria

Szwedo et Drohojowska **sp. nov.**; by present designation and monotypy.

#### Etymology

The generic name is derived from the adjective ‘dingla’ meaning ‘old’ in Jingpho language, which is spoken in Kachin state where the amber originates from. Gender: feminine.

#### Diagnosis

Vertex in mid line about as long as wide between compound eyes. Frons flat, widely triangularly incised at base. Antenna with 10th antennomere longer than penultimate one, widened, membranous apically, with terminal concavity. Pronotum about twice as wide as long. Mesopraescutum narrow, about as wide as pronotum; mesoscutum wide, with scutellar sutures not reaching anterior margin; mesoscutellum widely pentagonal. Fore wing with branch R forked anteriad of branch MP + CuA forking. Tip of clavus at level of MP + CuA forking. Hind wing with terminals RP and M subparallel and weakened in apical portion. Metafemur not thickened, metatibia without apical spines.

### *Dingla shagria* Szwedo & Drohojowska sp. nov

**Figure 2:**
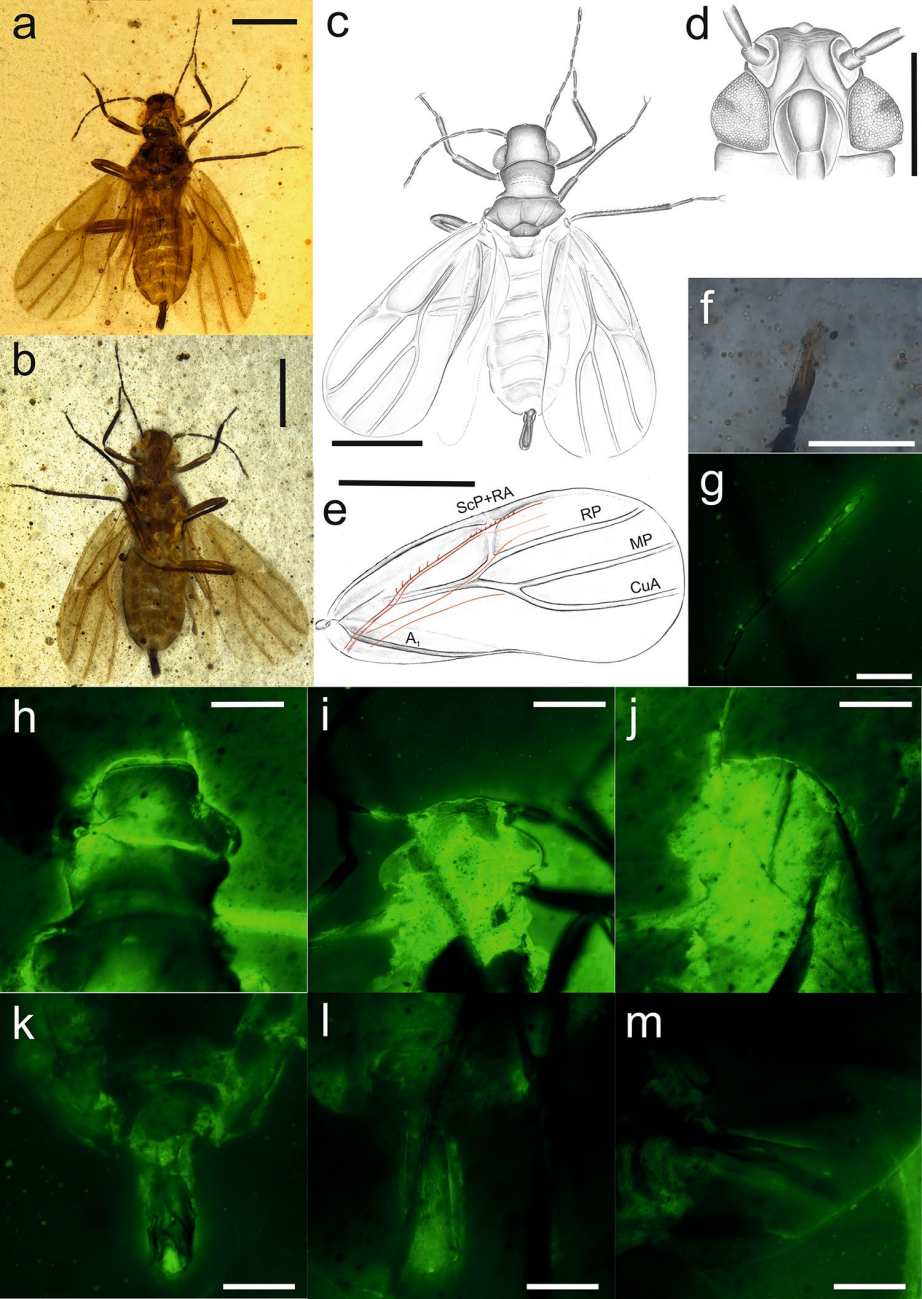
*Dingla shagria* gen. sp. nov., holotype male, No. MAIG 5,979: body in dorsal view (**a**); body in ventral view (**b**); drawing of body in dorsal view (**c**); drawing of head in ventral view with clypeus (**d**); fore wing (**e**); apical antennomere (**f**); antennomeres 6^th^—10^th^ (**g**); head in dorsal view (**h**); head in ventral view (**i**); head in lateral view (**j**); male genitalia in dorsal view (**k**); male genitalia in ventral view (**l**); male genitalia in lateral view (**m**); scale bars: 0.5 mm a, b, c, e; 0.1 mm f, g, k, l, m; 0.2 mm j, h, i; 0.25 mm d.

**Figure 3:**
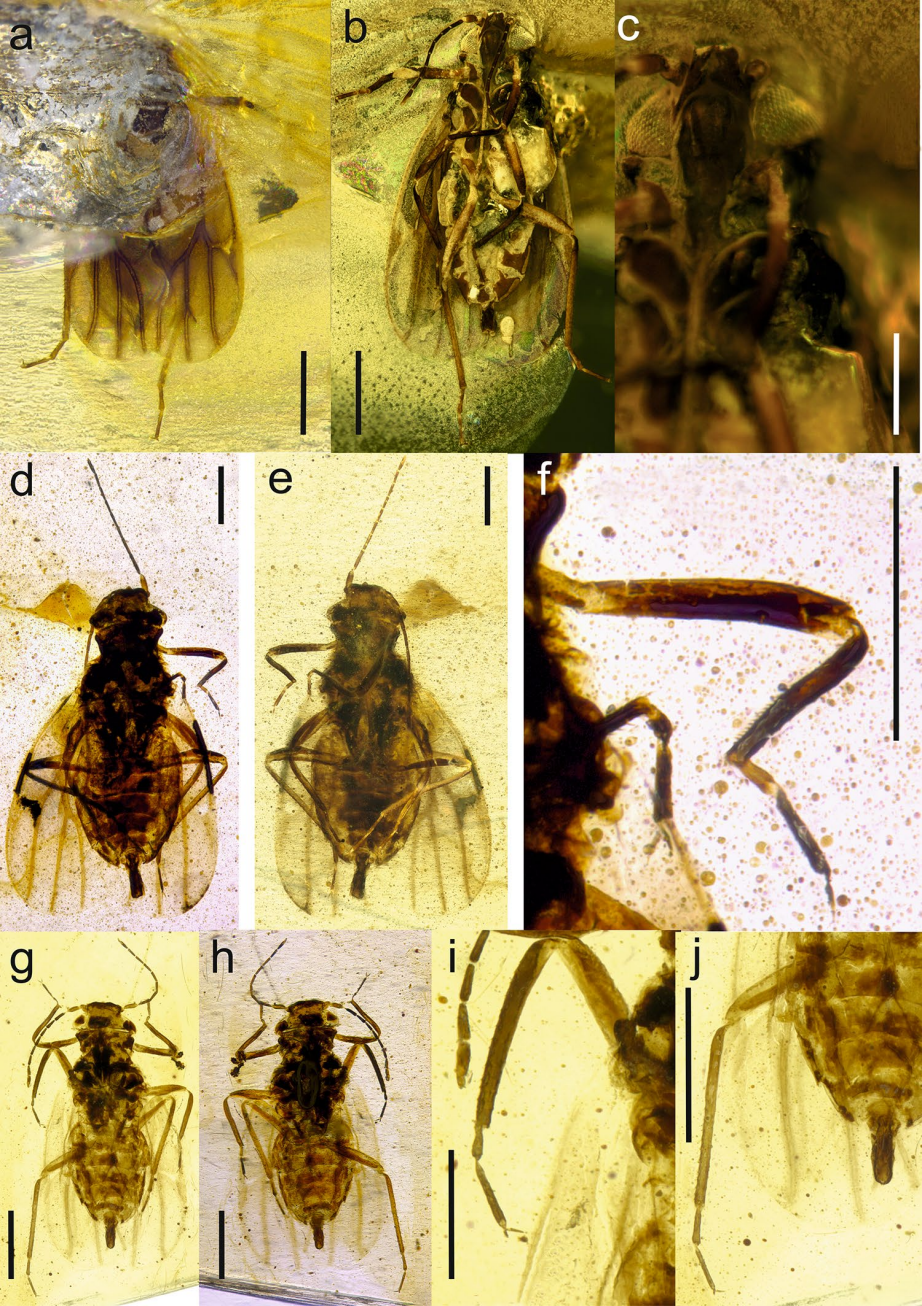
*Dingla shagria* gen. sp. nov., paratype male, No. MAIG 5,980: body in dorsal view (**a**); body in ventral view (**b**); head in ventral view with median ocellus (**c**); paratype male, No. NIGP172398, body in dorsal view (**d**); body in ventral view (**e**); fore tibia (**f**); paratype male, No. NIGP172399 body in dorsal view (**g**); body in ventral view (**h**); mid leg (**i**); hind leg (**j**); scale bars: 0.5 mm a, b, g, h, j; 0.4 mm d, e, f; 0.2 mm c; 0.25 mm i.

LSID urn:lsid:zoobank.org:act:3EA05FB0-B783-4D7A-98EA-10B02F50B83D (Figures 2, 3).

#### Etymology

The specific epithet is derived from the noun ‘shagri’ meaning ‘insect’ in Jingpho language spoken in the Kachin State, when the amber was collected.

#### Material

Holotype male. MAIG 5979, Paratype male, MAIG 5980, deposited in Museum of Amber Inclusions, Laboratory of Evolutionary Entomology and Museum of amber Inclusions, Department of Invertebrate Zoology and Parasitology, Faculty of Biology, University of Gdańsk, Gdańsk, Poland; paratype male NIGP172398, paratype male NIGP172399, deposited in Nanjing Institute of Geology and Palaeontology, Chinese Academy of Sciences, Nanjing, China.

#### Locality and horizon

Kachin amber, Noije Bum hill, Hukawng Valley, Kachin State, northern Myanmar. Terminal Aptian/earliest Cenomanian.

#### Diagnosis

Pedicel (2nd antennomere) elongate, slightly thickened, 3rd antennomere longer than second and 4th; antennomeres 4th to 8th subequal in length. Protibia with row of thin setae in apicad half. Probasitarsomere about half as long as prodistitarsomere. Subgenital plate small, subquadrate, parameres long and narrow, parallel; about 3 times as long as wide at base, with hooked acute apex. Male anal tube tubular, slightly widening apicad, merely shorter than parameres.

#### Description

Male. Measurements (in mm): Total length 1.76 to 2.13; Body length total (including claspers) 1.76–2.13; Head including compound eyes width 0.37–0.52; head length along mid line 0.18–0.24; vertex width 0.2–0.26; Forewing length 1.32–1.79; forewing width 0.62–0.74; Claspers length 0.2–032; Antennomere 1st 0.04–0.08; antennomere 2nd 0.8–0.13; antennomere 3rd 0.08–0.16; antennomere 4th 0.06–0.12; antennomere 5th 0.06–0.09; antennomere 6th 0.06–0.1; antennomere 7th 0.06–0.09; antennomere 8th 0.0–0.09; antennomere 9th 0.06–0.09; antennomere 10th 0.0.8–0.01; Profemur + protrochanter cumulative length 0.26–0.46; protibia length 0.29–0.34; probasitarsomere length 0.06–0.09; prodistitarsomere length 0.08–0.13; mesofemur + mesotrochanter cumulative length 0.3–0.4; mesotibia length 0.36–0.4; mesobasitarsomere length 0.05–0.1; mesodistitarsomere length 0.13–015; metafemur + metatrochanter cumulative length 0.39–0.56; metatibia length 0.5–0.68; metabasitarsomere length 0.1–0.15; metadistitarsomere length 0.1–0.18.

Vertex about half as long as width of head with compound eyes; slightly narrower than wide at base; disc of vertex slightly concave; sutura coronalis absent. Scapus cyllindrical, longer than wide, pedicel slightly longer than scapus, barrel-shaped, wider than 3rd antennomere. Antennomere 3rd longer than 2nd antennomere (pedicel) antennomeres 5th to 9th subequal in length; antennomere 9th with subapical rhinarium; antennomere 10th (apical) longer then penultimate one, spoon-like widened apically, with rhinarium placed subapically. Median and lateral ocelli visible from above. Compound eyes large, not divided, with distinct, non-differentiated ommatidia; postocular protuberances narrow. Frons convex, with distinct triangular, concave median portion; median ocellus at margin with vertex; postclypeus and apical portion of loral plates distinctly incised to frons; postclypeus about twice as long as wide; anteclypeus tapering ventrad; lora semicircular, long, with upper angles slightly below upper margin of postclypeus, lower angles not exceeding half of anteclypeus length. Rostrum with apex reaching metacoxae; scapus short, wide, placed in distinct anterolateral concavity.

Pronotum large, as long lateral as in midline; about 2.6 times as wide as long in mid line; disc of pronotum convex; anterior margin convex, slightly protruding between compound eyes; posterior margins converging posteriad; posterior margin slightly concave. Mesopraescutum with anterior margin covered by pronotum, with anterior margin convex, lateral margins expanded posterolaterad, with posterior margin convex posteriomediad, slightly concave posterolaterad. Mesoscutum distinctly wider than long in mid line; anterior margin merely concave medially, lateral margins distinctly diverging posteriad, posterolateral angles acute, distinct, posterior margin W-shaped, with distinct median concavity; disc of mesoscutum convex with indistinct longitudinal concavities (apodemes? sutures?). Mesoscutellum narrow, with anterior margin acutely convex, lateral margins subparallel, posterior margin straight, disc of mesoscutellum concave, with posteromedian furrow. Metascutum and metascutellum not visible.

Fore wing about 2.5 times as long as wide; narrower at base, widening posteriad, rounded in apical margin; widest at ¾ of its length. Costal margin thickened, veins thick, distinctly elevated; basal portion of stem R + MP + CuA weak, distal portion of stem R + MP + CuA convex, forked at about half of forewing length, branch RA short; pterostigmal area thickened; common stem MP + CuA short, branches RP, MP and CuA parallel on membrane; areola postica absent; clavus present, with apex exceeding half of forewing, with single claval vein A_1_.

Hind wing about 0.8 times as long as forewing, with costal margin with two groups of regularly dispersed setae, basal group with seven longer and stiff setae and median group with 10 shorter, stout setae; terminals RP and M subparallel and weakened in apical portion.

Profemur and mesofemur subequal in length; protibia slightly shorter than mesotibia; pro- and metadistitarsomeres slightly longer than pro- and mesobasitarsomeres. Metacoxa without meracanthus; metafemur longer than pro- and mesofemur; metatibia distinctly longer than pro- and mesotibia; metadistitarsomere distinctly longer than metabasitarsomere; tarsal claws long, narrow, without arolium or empodium.

Abdomen with segments III to VIII almost homonomic in length, widely connected to thorax, subgenital portion narrowing. Subgenital plate small, subquadrate, parameres long and narrow, parallel; about 3 times as long as wide at base, with hooked acute apex. Male anal tube tubular, slightly widening apicad, merely shorter than parameres.

## Discussion

**Figure 4:**
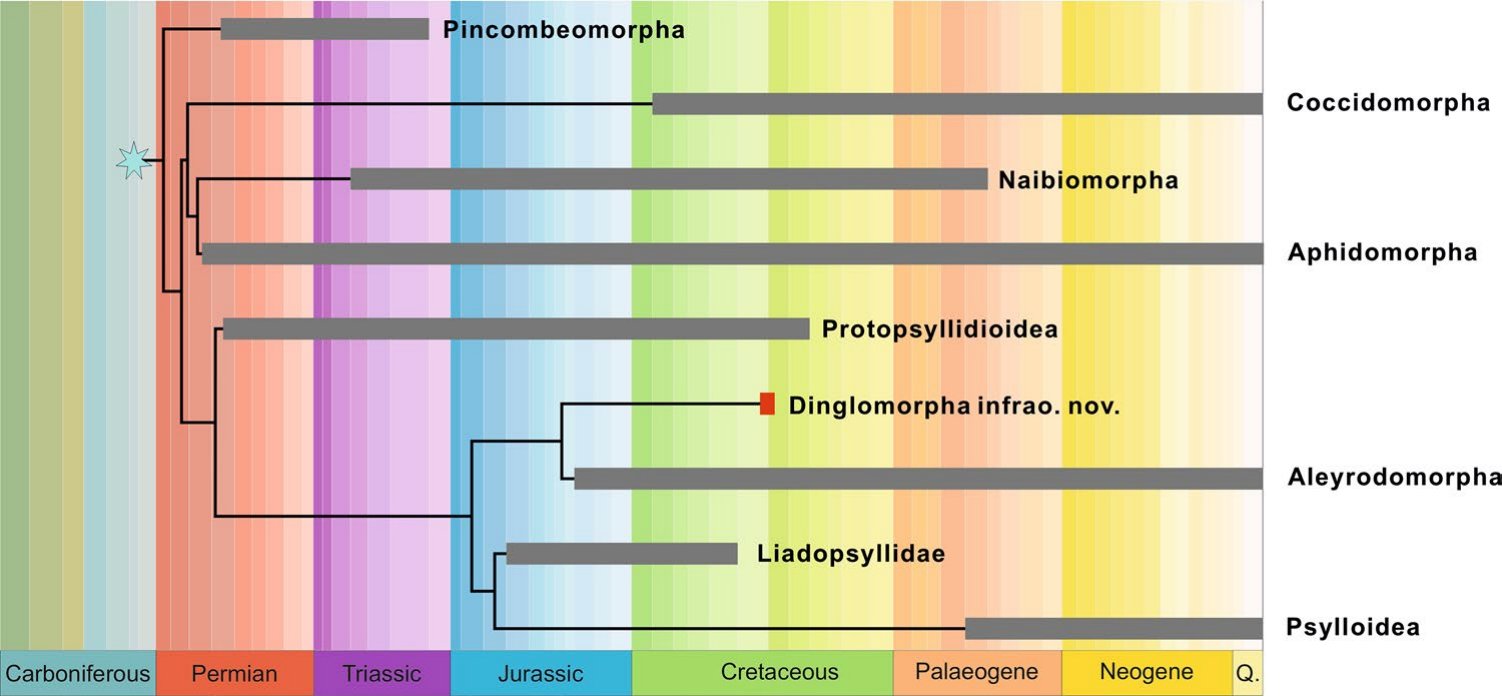
Chronophylogram of the main Sternorrhyncha lineages, based on the results of present analyses.

Dinglomorpha **infraord. nov.** forms a distinct group, nested within a clade of Psylliformes, related to Aleyrodomorpha: Aleyrodoidea and Psyllodea: Psylloidea, but deserving to be separated as a different infraorder (Figs. 1, 4). This new infraorder seems to be closer related, in terms of its morphological features, to Psylliformes, the group containing Protopsyllidioidea, Aleyrodomorpha, extinct Liadopsyllidae and modern Psylloidea. Dinglomorpha **infraord. nov.** shares some features with Aleyrodomorpha, e.g. the general structure of head capsule, retention of antennal processus terminalis, membranous mesoscutellum, well developed mesopostnotum, and in fore wing venation reduction of areola postica. Dinglomorpha presents a combination of unique features, such as vein ScP present as separate fold at the base of common stem R + MP + CuA and base of this stem weakened (this feature is autapomorphic for the group and not observable in any other Sternorrhyncha). The presence of 10 antennal segments (antennomeres) seems to be a very conservative feature, as a reduction of the number of antennomeres is the general morphological tendency observed in various sternorrhynchans^15^. The presence of a median ocellus directed anteriorly seems to be a symplesiomorphic condition retained in some basal sternorrhynchans, e.g. in Jurassic Liadopsyllidae, Cretaceous genera *Yamis* Drohojowska & Szwedo, 2015 and *Shapashe* Drohojowska & Szwedo, 2015 (Aleyrodidae), or Cretaceous Postopsyllidiidae^10,11,13,16^.

The general structure of the head capsule in *Dingla* **gen. nov.** partly resembles the pattern observed in Psylloidea, with a narrow frontal portion incised between enlarged genae^17^. On the other hand, the well developed postclypeus and anteclypeus, with large mandibular plates (lora), and bases of antennae placed distinctly in front of compound eye resemble the pattern present in Aleyrodidae^18^. The antennae of *Dingla* **gen. nov.** have rhinaria on the ultimate and penultimate antennomeres, which is different than in other Psylliformes. In Psylloidea rhinaria are present subapically on each of antennomeres 2, 4, 6, and 7, in the Aphalarinae, rhinaria are also present on antennomeres 3rd and 5th^19^, in Protopsyllididae rhinaria seems to be distributed on antennomeres 3rd to 10th. It is not clear in Postopsyliididae and Permopsyllididae^11,13^, but it is most probably the same as in Protopsyllididae. In recent species of Aleyrodidae, rhinaria are usually present on antennomeres 3rd, 5th, and 7th^20^, however, multiple rhinaria are known in extinct *Gapenus rhinariatus* Drohojowska & Szwedo, 2013 from the Lower Cretaceous Lebanese amber^21^.

The pronotum in *Dingla* **gen. nov.** is relatively large, most similar to the state in *Postopsyllidium* Grimaldi, 2003 (Postopsyllidiidae; see^13^). In general appearance it is similar to the pronotum observed in other Psyllodea, however it is larger than in Psylloidea and Aleyrodoidea^14,17,18^. The mesopraescutum in Dinglomorpha **infraord. nov.** is narrow, partly covered by the posterior portion of the pronotum. In Aleyrodoidea the mesopraescutum is not covered by the pronotum, with the posterior margin angulate, incised to the mesoscutum^14,18^. In general appearance it is most similar in shape to the the mesocutum in Psylloidea^14^. The mesopraescutum is poorly known in Protopsyllidioidea. It is relatively small, diamond shaped and with the anterior portion covered under the pronotum in *Postopsyllidium* Grimaldi, 2003^13^. The mesoscutum of *Dingla* **gen. nov.** is quite large, as in Psylloidea and Aleyrodidae, but has a deep posterior incision in which the mesoscutellum is incised with its anterior portion. In Aleyrodidae the mesoscutellum is short, membranous, and its median portion could be incised in posterior margin of mesoscutum^18,21–25^. The well-developed mesopostnotum is present in *Dingla* **gen. nov.** and Aleyrodidae, while it is not as distinct in Psylloidea^14,17^. The metascutum, metascutellum and metapostnotum in *Dingla* **gen. nov.** are poorly visible, probably less developed in comparison to Psylloidea or Aleyrodoidea^14,17,18^.

Venation of the fore wing in Dinglomorpha **infraord. nov.** is very peculiar. The costal margin is thickened, with carinate Pc, as in remaining Psyllodea. The costal break, characteristic of Psylloidea is missing here, however, the veins of costal complex are at least partly included in thickened ambient vein—this vein is well developed in Psylloidea. The structure of the basal portion of the fore wing in Dinglomorpha is very unusual—the basal portion of veins R + MP + CuA is weakened, with ScP separated as fold. The median portion of R + MP + CuA complex presents traces of independence of stem R and stem MP + CuA, the fork of this stem is placed basal of claval apex, stem R produces single RA and much longer RP. The homologisation of the second branch is uncertain—from the topographic position on the wing it seems more probable that it is an MP stem, and that CuA, with its fork (delimiting the areola postica), is reduced. A similar reduction of areola postica is observed in Aleyrodidae, but in whiteflies the MP stem is also reduced (weak or absent in extinct Bernaeinae; see^26,27^), or absent in Aleyrodinae and Aleurodicinae^23^.

The early stages of Sternorrhyncha evolution are not well understood, which is reflected in doubts and incongruences in their classification hypotheses based on morphological, palaeontological and molecular data (Fig. 4). The classification and nomenclatorial history of the Sternorrhyncha is very complex^28^ (see also Supplementary information 1). The division of the Sternorrhyncha into two independent lineages was already postulated by Börner^29^, leading to opinions of non-monophyletic (diphyletic) status of the suborder^26^. Those proposals result from palaeontological observations and interpretations of the independent origins of aphids + scale insects lineage and jumping plantlice + whiteflies lineage, as well as inclusion of Paleorrhyncha (paraphyletic Archescytinoidea) within Sternorrhyncha^12^.

Morphological characters supporting the monophyly of the Sternorrhyncha comprises the rostrum tightly attached to chest, mesonotum divided into sclerites (unknown state in Pincombeomorpha), and reduced (in vast majority) veinlet cua-cup at base of fore wing. Development of the stigmal area in Pincombeomorpha and Aphidomorpha + Naibiomorpha appears to be homoplastic, however this feature could be a local synapomorphy of this lineage. Numerous morphological details of extinct Pincombeomorpha are poorly known. In most cases only isolated wings are available as sources of data. Regarding venational patterns, Pincombeidae seems to be more similar to the Aphidomorpha + Coccidomorpha lineage^30,31^. The analysis of head and thorax structures presented by Wegierek^32^ shows that the Aleyrodomorpha displays a set of apomorphies which are not found in other groups of Sternorrhyncha, and these features place Aleyrodomorpha as a sister group to other sternorrhynchans, but in an unresolved position with regards to Euhemiptera (Supplementary information 1 Fig. S4d). Molecular studies are incongruent with the fossil record and morphological analyses, postulating Sternorrhyncha as a monophylum (Supplementary information 1 Fig. S3a–c), a sister group to remaining hemipterans^1^. Molecular studies often place Aleyrodomorpha as sister group to other Sternorrhyncha^33–35^, while results of morphological analyses suggest Aleyrodomorpha as a sister group to Psyllodea^14,36^. See also Supplementary information 1 for more detailed comments on relationships within the Sternorrhyncha.

The oldest fossils ascribed to the Sternorrhyncha were recently reported from the Moscovian (Carboniferous) locality of Avion in Pas-de-Calais Basin, France^5^. This finding pushes back the history of the group (Fig. 4) and challenges the hypothesis of their direct descendance from the Paleorrhyncha Archescytinoidea, which are known from the Asselian (earliest Permian) as previously proposed^26,32^.

The fossil record of particular sternorrhynchan lineages and their diversification, palaeodiversity and palaeodisparity is very uneven (see Supplementary information 1). Early diversity of Psyllodea comprises various Protopsyllidioidea, which went extinct by the late Cretaceous^13^. Jurassic diversity of modern Psylloidea and Aleyrodoidea is poorly documented, however their diversification might have been hampered by competition from other sternorrhychans and phloem-feeders radiating at these times (planthoppers and some true-bugs). Jurassic Liadopsyllidae present many plesiomorphic conditions, suggesting that these insects were still very generalized in their morphology and not highly disparate, as observed among other sternorrhynchans. The morphology of the Aleyrodidae adults is also rather conservative and not highly disparate as we can observe from the Jurassic and Cretaceous fossils^25,27^. The evolutionary shift in the morphological disparity of whiteflies (their puparia, in fact) is most probably related to mid-Cretaceous biosphere reorganization^37^, resulting in the change of host plants from gymnosperms to angiosperms and co-radiation with them. The evolutionary scenario of Dinglomorpha **infraord. nov.** was probably also affected by these and, as result, these insects could be endemic to the mid-Cretaceous biota of Kachin amber forests, as has been observed among other insects^7^. The distinctness of Dinglomorpha **infraord. nov.** could be a result of their long, alas so far undocumented, evolutionary history on the West Burma terrane or even Gondwanaland. The geological history of this terrane is very complex^38–40^. The West Burma terrane (West Burma block) separated from Australia in the Late Jurassic^41,42^. The placement of West Burma Block in the Cretaceous is a subject of numerous discussions^42^, and the question of whether West Burma was originally a part of Sibumasu or a part of the Lhasa Block still remains open. Various interpretations of tectonics led to a complex series of various palaeobiogeographic scenarios, relating the Burmese amber fauna with Gondwanan elements^43,44^. On the other hand, numerous groups known from slightly older Palaeoasian fossil sites are present among Kachin amber inclusions as well^7^. Among the Sternorrhyncha families, there is no clear distributional pattern; families distributed more widely in the Lower Cretaceous to the times of Kachin amber formation, as well as families known so far exclusively from Kachin amber were reported. Aphids of the families Burmitaphididae, Juraphididae, Szelegiewicziidae and Tajmyraphidiidae are reported from several Lower Cretaceous fossil sites. Only the family Parvaverrucosidae is unique for Kachin amber. The scale insect families Coccidae, Hodgsonicoccidae, Margarodidae, Matsucoccidae, and Xylococcidae are known from various Lower Cretaceous sites, while Cretaceous records of Kozariide, Ortheziidae, Pseudococcidae, and Weitschatidae are unique from Kachin amber. Postopsyllidiidae are known from Kachin amber and from Turonian Raritan amber of New Jersey (U.S.A.). Aleyrodidae were reported from Lower Cretaceous Lebanese amber and Mongolia, and from Kachin amber as well. Dinglidae **fam. nov**. (Dinglomorpha **infraord. nov.**) for the moment are exclusively known from Kachin amber. Morphological disparity of Dinglomorpha, clearly separating this lineage from the other relatives, together with its limited distribution, could support the Gondwanan influence on the composition of the Kachin amber inclusions.

## Conclusions

We described a new genus and species, representing a peculiar and disparate sternorrhynchan lineage, known so far only from Kachin amber. It extends the range of the known taxonomic diversity and morphological disparity of Sternorrhyncha. Its morphological characters led to the placement of Dinglomorpha as separate infraorder, sister to Aleyrodomorpha (Psylliformes). The morphological disparity of Dinglomorpha could be due to their isolation and separate evolutionary history on the West Burma terrane, which seems to have been influenced by ecological pressures and challenges related to the local biota. The features and fate of the fossils preserved in Kachin amber were shaped by major ecological changes during the Cretaceous, making Dinglomorpha an example of a highly specialized, short-lived lineage of the Sternorrhyncha.

The results of the first phylogenetic analysis of all sternorrhynchan groups, which is presented here, confirmed the monophyly of Sternorrhyncha, revealed Pincombeomorpha as a sister group to the remaining lineages, and supported the hypothesis of separating them into two clades – Aphidiformes and Psylliformes. The finding described above gives additional insight into the systematics, diversity and disparity of the Sternorrhyncha. The palaeoecology of the new group seems to be related to tropical habitats of the West Burma terrane, at least since the time of its separation from Australia in the Late Jurassic. Dinglomorpha could be one of the groups of Gondwanan origin and therefore the finding is also important for understanding the palaeobiogeography and the evolutionary history of the fauna of the Kachin amber forest.

## Material and methods

The studied specimens are inclusions in mid-Cretaceous amber from Burma (Myanmar). Two specimens were collected by Mr. Patrick Müller, and acquired by the Museum of Amber Inclusions, University of Gdańsk (MAIUG) and two more come from the collection of Nanjing Institute of Geology and Palaeontology, Chinese Academy of Sciences (NIGPAS). Specimens were cut, grinded and polished for better visibility.

The specimens were examined, photographed and measured using the Leica M205C, Nikon SMZ1500, Nikon SMZ1270, Nikon Eclipse E600 and Zeiss Axio.Imager digital microscopes platforms, with incident and transmitted light were used simultaneously as well as with fluorescent illumination. The illustrations were prepared with two image-editing software packages (CorelDraw X9, CorelPaintX9). Fourier Transform Infrared Spectra (Supplementary information 1 Fig. S3a-h) were obtained in the Amber Laboratory of the International Amber Association in Gdańsk, for the reasons and according to procedure proposed by Szwedo and Stroiński^45^.

Phylogenetic analyses were performed according to procedures described in Supplementary information 1. Matrix file is presented as Supplementary information 2.

## Supplementary information

Supplementary Information 1.

Supplementary Information 2.

## References

[1] Szwedo J (2018). The unity, diversity and conformity of bugs (Hemiptera) through time. Earth Environ. Sci. Trans. R. Soc..

[2] Henry TJ, Foottit AG, Adler PH (2017). Biodiversity of Heteroptera. Insect Biodiversity Science and Society.

[3] Bartlett CR, Foottit AG, Adler PH (2018). The diversity of the true hoppers (Hemiptera: Auchenorrhyncha). Insect Biodiversity. Science and Society.

[4] Hardy NB, Foottit AG, Adler PH (2018). The biodiversity of Sternorrhyncha: scale insects, aphids, psyllids, and whiteflies. Insect Biodiversity. Science and Society.

[5] Garrouste, R., Schubnel, T., Oudard, J., Roques, P. & Nel, A. The insect Konservat-Lagerstätte of the Upper Carboniferous of Avion (France): an exceptional geoheritage. in *Abstracts. 8*^*th*^* International Conference on Fossil Insects, Arthropods & Amber, Santo Domingo 2019* (ed. Nascimbene, P.C.) 43–44 (Amber World Museum, Santo Domingo 2019).

[6] Wang YH (2016). Fossil record of stem groups employed in evaluating the chronogram of insects (Arthropoda: Hexapoda). Sci. Rep..

[7] Ross AJ (2019). Burmese (Myanmar) amber checklist and bibliography 2018. Palaeoentomology.

[8] Heie OE, Wegierek P (2011). A list of fossil aphids (Hemiptera, Sternorrhyncha, Aphidomorpha). Monogr. up. siles. Mus..

[9] Shcherbakov DE (2007). Extinct four-winged precoccids and the ancestry of scale insects and aphids (Hemiptera). Russ. Entomol. J..

[10] Grimaldi DA (2003). First amber fossils of the extinct family Protopsyllidiidae, and their phylogenetic significance among Hemiptera. Ins. Syst. Evol..

[11] Becker-Migdisova EE (1985). Iskopaemye nasekomye psillomorfy [Fossil psyllomorphan insects]. Trudy Paleontol. Inst..

[12] Klimaszewski SM, Wojciechowski W (1992). Relationships of recent and fossil groups of Sternorrhyncha as indicated by the structures of their forewings. Prace nauk. Uniw. Śląskiego.

[13] Hakim M, Azar D, Szwedo J, Brysz AM, Huang DY (2019). New paraneopterans (Protopsyllidioidea, Hemiptera) from the mid-Cretaceous amber of northern Myanmar. Cret. Res..

[14] Drohojowska J (2015). Thorax morphology and its importance in establishing relationships within Psylloidea (Hemiptera, Sternorrhyncha). Prace nauk. Uniw. Śląskiego.

[15] Shaposhnikov, G.Kh. Oligomerizatsiya, polimerizatsiya i uporyadochenie morfologicheskikh struktur v evolyutsii tleï (Homoptera, Aphidinea). *Entomol. Obozr*. **58** (4), 716–741 (1979). (Published in English as: Shaposhnikov, G. Kh. The oligomerization, polymerization, and ordering of morphological structure in the evolution of aphids (Homoptera, Aphidiniea). *Entomol. Rev.* **59**, 27–52 (1980)).

[16] Drohojowska J, Szwedo J (2015). Early Cretaceous Aleyrodidae (Hemiptera: Sternorrhyncha) from the Lebanese amber. Cret. Res..

[17] Weber H (1929). Kopf und Thorax von Psylla mali Schmidb. (Hemiptera-Homoptera) Eine morphogenetische Studie. Z. Morphol. Ökol. Tiere.

[18] Weber H (1935). Der Bau der Imago der Aleurodinen Ein Beitrag zur vergleichenden Morphologie des Insektenkorpers. Zoologica.

[19] Ossiannilsson F (1992). The Psylloidea (Homoptera) of Fennoscandia and Denmark. Fauna Entomol. Scand..

[20] Gill RJ, Gerling D (1990). The morphology of whiteflies. Whiteflies: Their Bionomics, Pest Status and Management.

[21] Drohojowska J, Szwedo J, Azar D (2013). Gapenus rhinariatus gen. sp. n. from the Lower Cretaceous amber of Lebanon (Hemiptera Sternorrhyncha Aleyrodidae). Insect Evolution in an amberiferous and stone alphabet. Proceedings of the 6th International Congress on Fossil Insects, Arthropods and Amber.

[22] Drohojowska J, Szwedo J (2011). A new whitefly from Lower Cretaceous Lebanese amber (Hemiptera: Sternorrhyncha: Aleyrodidae). Inst. Syst. Evol..

[23] Drohojowska J, Szwedo J (2013). The first Aleyrodidae from the Lowermost Eocene Oise amber (Hemiptera: Sternorrhyncha). Zootaxa.

[24] Drohojowska J, Perkovsky EE, Szwedo J (2015). New genus and species of Aleyrodidae from the Eocene Baltic amber (Hemiptera: Sternorrhyncha: Aleyrodomorpha). Pol. J. Entomol..

[25] Szwedo J, Drohojowska J (2016). A swarm of whiteflies—the first record of gregarious behavior from Eocene Baltic amber. Sci. Nat..

[26] Shcherbakov DE (2000). The most primitive whiteflies (Hemiptera; Aleyrodidae; Bernaeinae subfam. nov.) from the Mesozoic of Asia and Burmese amber, with an overview of Burmese amber hemipterans. Bull. Nat. Hist. Mus. Lond. (Geol.).

[27] Drohojowska J, Wegierek P, Evans GA, Huang DY (2019). Are contemporary whiteflies “living fossils”? Morphology and systematic status of the oldest representatives of the Middle-Late Jurassic Aleyrodomorpha (Sternorrhyncha, Hemiptera) from Daohugou. Palaeoentomology.

[28] Kluge NYu (2010). Circumscriptional names of higher taxa in Hexapoda. Bionomina.

[29] Börner C (1904). Zur Systematik der Hexapoden. Zool. Anz..

[30] Szwedo J, Lapeyrie J, Nel A (2015). Rooting down the aphid’s tree – the oldest record of the Aphidomorpha lineage from Palaeozoic (Insecta: Hemiptera). Syst. Entomol..

[31] Szwedo J, Weis R, Nel A (2019). A bizarre sternorrhynchan wing from the Lower Jurassic of Luxembourg (Hemiptera: Sternorrhyncha: Pincombeomorpha?). Hist. Biol..

[32] Wegierek P (2002). Relationships within Aphidomorpha on the basis of thorax morphology. Prace nauk. Uniw. Śląskiego.

[33] Misof B (2014). Phylogenomics resolves the timing and pattern of insect evolution. Science.

[34] Johnson KP (2018). Phylogenomics and the evolution of hemipteroid insects. Proc. Natl. Acad. Sci. USA.

[35] Kieran TJ (2019). Insight from an ultraconserved element bait set designed for hemipteran phylogenetics integrated with genomic resources. Mol. Phyl. Evol..

[36] Schlee D (1969). Sperma-Übertragung (und andere Merkmale) in ihrer Bedeutung für das phylogenetische System der Sternorrhyncha (Insecta, Hemiptera). Phylogenetische Studien an Hemiptera. 1. Psylliformes (Psyllina and Aleyrodina) als monophyletische Gruppe. Z. Morphol. Tiere.

[37] Szwedo J, Nel A (2015). The Cretaceous insects: a promising state of the art. Cret. Res..

[38] Searle MP (2017). Tectonic and metamorphic evolution of the Mogok Metamorphic and Jade Mines belts and ophiolitic terranes of Burma (Myanmar). Geol. Soc. London Mem..

[39] Barber AJ, Zaw K, Crow MJ (2017). The pre-Cenozoic tectonic evolution of Myanmar. Geol. Soc. London Mem..

[40] Morley CK, Naing TT, Searle M, Robinson SA (2020). Structural and tectonic development of the Indo-Burma ranges. Earth-Sci. Rev..

[41] Heine C, Müller RD, Gaina C (2004). Reconstructing the lost Eastern Tethys Ocean basin: convergence history of the SE Asian margin and marine gateways. Geophys. Monogr. Ser..

[42] Metcalfe I (2017). Tectonic evolution of Sundaland. Bull. Geol. Soc. Malays..

[43] Xing XL (2018). A mid-Cretaceous embryonic-to-neonate snake in amber from Myanmar. Sci. Adv..

[44] Poinar G (2019). Burmese amber: evidence of Gondwanan origin and Cretaceous dispersion. Hist. Biol..

[45] Szwedo J, Stroiński A (2017). Who’s that girl? The singular Tropiduchidae planthopper from the Eocene Baltic amber (Hemiptera: Fulgoromorpha). Palaeontol. Electronica.

[46] Szwedo J, Nel A (2011). The oldest aphid insect from the Middle Triassic of the Vosges France. Acta Palaeontol. Pol..

[47] Yang G, Yao YZ, Ren D (2012). A new species of Protopsyllidiidae (Hemiptera, Sternorrhyncha) from the Middle Jurassic of China. Zootaxa.

[48] Ouvrard D, Burckhardt D, Azar D, Grimaldi D (2010). Non-jumping plant-lice in Cretaceous amber (Hemoptera: Sternorrhyncha: Psylloidea). Syst. Entomol..

