# Supplementary material for: Fossils reshape the Sternorrhyncha evolutionary tree (Insecta, Hemiptera)
Source: Sci Rep. 2020 Jul 9;10:11390. doi: 10.1038/s41598-020-68220-x (PMC7347605; doi:10.1038/s41598-020-68220-x)
Supplement: Supplementary file 1 — Supplementary Information 1. [file 41598_2020_68220_MOESM1_ESM.pdf]

Drohojowska, Szwedo, Żyła, Huang & Müller: Fossils reshape the Sternorrhyncha evolutionary tree (Insecta, Hemiptera)

Jowita Drohojowska, Jacek Szwedo, Dagmara Żyła, Di-Ying Huang & Patrick Müller

Supplement 1

1. Geological setting

The specimens described here come from the Noije Bum amber deposit in the Hukawng Valley in Kachin State, northern Myanmar fig. 1[49,50]. The age of the Kachin amber was determined as Turonian-Cenomanian based on arthropods[51]. Due to the discovery of the ammonite *Mortoniceras* Meek, 1876 it was stated to be Middle Albian-Upper Albian[52], and the age dating of 98.79 ± 0.62 Ma was determined by Shi *et al.*[53]. However, a slightly older, late Aptian age of amber was recently postulated[54], due to the fact that the amber shows evidence of redeposition[55,56]. A recent finding of an ammonite inclusion in this resin[57], did not end the debates about its age. Burmese amber, mineralogical named as burmite by Gdańsk pharmacist Otto Helm[58,59,60], until the end of the last century was regarded as rare and weakly known fossil resin. The interest in burmite and its inclusions exploded during the past two decades and resulted in the description of hundreds of taxa from this amber[7]. Burmese amber preserves enormous diversity of plants, invertebrates and vertebrates[7], giving new insight into a very important period of formation of modern faunistic complexes at the times of mid-Cretaceous biotic re-organisation[37]. Burmese amber was proposed to be a derivative of the resin exuded from the gymnosperm trees of family Araucariaceae[61] but recently the Cupressaceae, with *Metasequoia* were proposed as source-plants for this resin[55,62]. The Sibumasu terrane, which might be the nearest landmass of the locality where burmite was deposited, was placed in the climatic tropical zone[63,64]. Palynological study also suggested a humid, warm-temperate climate[52], indicating the presence of an equatorial floristic realm.

Sediments that host the burmite are a variety of clastic sedimentary rocks, with thin limestone beds, and abundant coaly and carbonaceous material, and amber is found within a narrow horizon in the fine clastic facies. Accompanying the records of macrofossils like ammonite, gastropods and bivalves, and the microfauna including dinoflagellates, the depositional environment was suggested to be a nearshore marine setting close to deltas[40,65,66]. The amber locality lies within the West Burma terrane[40,67,68], which finally collided with the Eurasian marginal Sibumasu terrane at around 80 Ma[69,70], however, various unconstrained age

estimates are proposed ranging from the Middle Jurassic to the latest Cretaceous[71]. This suggests some island or archipelago environments for at least part of the amberiferous area at time of resin formation and deposition[40,43,68,72,73].

2. Morphological terminology

There is no consensus on the venation pattern and vein homology in Sternorrhyncha. Various authors[9,16,19, 21,22,23,74,75,76] have used various names and interpretations. Nel *et al.*[77] proposed a new interpretation of the wing venation pattern for all Paraneoptera, assuming that CuA gets fused with R+MP stem at wing base and connected with CuP by a specialized crossvein *cua-cup*, which is remarkably different from the traditional interpretations. An additional point is the presence of complete fusion of MA with R in Paraneoptera, so that only MP is present. The venational terminologies used herein are slightly modified from Nel *et al.*[77], while the nomenclature of body structures mainly follows Drohojowska & Szwedo[22]. Veins abbreviations used: Pc – precosta; ScP – subcosta posterior; R – radius; RA – radius anterior; RP – radius posterior; MP – media posterior; CuA – cubitus anterior; A_1 – analis primus (first anal).

3. Morphological characters

Morphological characters that are discernible in the fossils as well as extant taxa were selected for the phylogenetic analyses. The data matrix used for the analysis consists of 10 taxa (Fulgoromorpha taken as an outgroup, and 9 Sternorrhyncha ingroups, including extinct groups, see Supplement 1 Table S1) and 42 characters (see Supplement 1 Table S2). Unknown character states were coded with '?', while inapplicable states with '–'. The list of characters and the nexus file containing the character matrix is available in Supplement 1 (Tables S1 and S2). The matrix was prepared with Mesquite version 3.61[78]. The nexus file containing the character matrix is available as supplementary file Supplement 2.

Table S1. Matrix of characters

	1	2	3	4	5	6	7	8	9	10	11	12	13	14	15	16	17	18	19	20
Fulgoromorpha	0	0	0	1	1	1	1	0	0	0	0	0	0	-	-	-	-	-	-	0
Pincombeomorpha	?	0	?	?	?	?	?	0	0	0	1	0	?	?	?	?	?	?	?	?
Coccomorpha	1	0	0	0	0	1	1	1	0	0	1	1	1	0	0	0	0	1	1	1
Naibiomorpha	0	0	0	0	0	0	0	0	0	1	1	0	1	0	0	1	0	1	1	1

Aphidomorpha	0	0	0/1	0	0	0	0	0	0	1	1	0	1	0	0	1	0	1	0	1
Protopsyllidioidea	0	0	0/1	0	0	1	1	0	0	0	0	0	1	0	0	1	0	1	0	0
Liadopsyllidae	0	0	0	0	0	0	1	0	0	0	2	0	1	1	1	0	0	1	0	0
Psylloidea	0	1	0/1	0	0	0	1	0	0	0	2	0	1	1	1	0	0	1	0	0
Dinglomorpha	0	0	0	0	0	?	0	0	0	0	1	0	1	1	1	0	1	0	0	0
Aleyrodomorpha	0	0	0	0	0	0	0	0	1	0	1	0	1	1	1	0	1	0	0	0

	21	22	23	24	25	26	27	28	29	30	31	32	33	34	35	36
Fulgoromorpha	0	1	0	0	0	0	1	0	1	0	0	0/1	0	0	0	0
Pincombeomorpha	0	?	0	0	0	0/1	0	0	1	0	0	1	0	0	0	0
Coccomorpha	0	0	1	1	0	1	0	0	0	0	0	0	1	1	1	1
Naibiomorpha	0	0	1	1	0	1	0	0	0	0	0	1	1	1	0	1
Aphidomorpha	0	0	1	1	0	1	0	0	0	0	0	1	0	1	0	1
Protopsyllidioidea	0/1	0	?	0/1	1	0/1	1	0	0	1	0	0	0	0/1	0	0
Liadopsyllidae	0	0	1	1	1	1	1	0	1	1	0	0	0	1	0	0
Psylloidea	0	1	1	1	1	1	1	1	1	1	0	0	0	1	0	0
Dinglomorpha	0	0	1	1	1	1	1	0	0	1	1	0	1	1	1	0
Aleyrodomorpha	0	0	1	1	1	1	1	0	0	1	0	0	0	1	1	0

	37	38	39	40	41	42
Fulgoromorpha	0	0/1	0	0	0	0
Pincombeomorpha	0	0	0	0	?	?
Coccomorpha	0	0	1	1	0	0
Naibiomorpha	0	0	1	0	0	0
Aphidomorpha	0	0	1	0	0	0
Protopsyllidioidea	1	1	0	0	1	1
Liadopsyllidae	1	0	0	0	1	1
Psylloidea	1	1	0	0	1	1
Dinglomorpha	1	0	0	0	0	1
Aleyrodomorpha	1	0	0	0	1	1

Following morphological characters and their states were used for the analysis (Supplement Table S2).

Table S2. List of characters and their states.

Head:

1. Head capsule: 0 – uniform; 1 – divided into several sclerites

2. Genal cones: 0 – absent; 1 – developed
3. Setae on head: 0 – absent; 1 – present
4. Antennal flagellum: 0 – absent; 1 – present
5. Sensory structures on pedicel: 0 – absent; 1 – present
6. Rhinaria on antennae: 0 – present; 1 – absent
7. Antennal processus terminalis: 0 – present; 1 – absent
8. Imaginal compound eyes ommatidia: 0 – with regular ommatidia; 1 – modified up to single rows or groups
9. Imaginal compound eyes dorsoventrally: 0 – not divided; 1 – divided
10. Imaginal compound eye additional structures: 0 – absent; 1 – compound eye accompanied by additional ocellum
11. Rostrum: 0 – free, not depressed to chest; 1 – placed in sternal depression; 2 – fused with prothoracic sternite
12. Rostrum well developed: 0 – in all morphs; 1 – mouth parts reduced in males

Thorax:

13. Mesonotum: 0 – uniform, not divided; 1 – divided into several plates
14. Mesopraescutum: 0 – markedly smaller than mesoscutum; 1 – mesoscutum and meso-praescutum comparable in size
15. Mesopraescutum: 0 – surrounded by mesoscutum; 1 – mesopraescutum projected before mesoscutum
16. Mesoscutum: 0 – weakly convex; 1 – strongly raised mesoscutum forms two humps
17. Mesoscutellum: 0 – distinctly separate, strongly sclerotised; 1 – mesoscutellum reduced, membranous
18. Mesopostnotum: 0 – well developed; 1 – mesopostnotum poorly visible in dorsal view
19. Metathoracic tergite: 0 – divided into metanotum and metapostnotum; 1 – metathora-cic tergite in the form of vestigial sclerite

Legs:

20. Bases of legs: 0 – on the underside of body; 1 – bases of legs on the sides of body
21. Fore femora: 0 – naked; 1 – armed with long spines
22. Hind coxae: 0 – not large, not fused with sternum; 1 – large, fused with sternum
23. Tarsi: 0 – 3-segmented; 1 – 2 to 1 segmented

Fore wings:

24. Basal cell: 0 – present; 1 – absent
25. Vein Pc: 0 – not carinate; 1 – carinate

26. Veinlet cua-cup at basal cell: 0 – present; 1 – absent
27. Costal complex of veins: 0 – not thickened; 1 – thickened
28. Costal break: 0 – absent; 1 – present
29. Costal cell (between Pc+CA+CP and ScP+R): 0 – narrow; 1 – broadened
30. Vein ScP: 0 – long, reaching margin with R; 1 – short, not reaching margin
31. Very basal portion of common stem ScP+R+MP+CuA: 0 – distinct; 1 – reduced
32. Stigmal area: 0 – not thickened; 1 – thickened
33. Vein MP: 0 – forked; 1 – single
34. Cross vein rp-mp: 0 – present; 1 – absent
35. Areola postica: 0 – present; 1 – absent
36. Clavus: 0 – fully developed; 1 – reduced to absent
37. Thickened ambient vein: 0 – absent; 1 – present
38. Longitudinal veins of fore wings covered with setae: 0 – no; 1 – yes

Hind wings:

39. Hind wings: 0 – fully developed, longer than 2/3 of fore wing; 1 – reduced in size, shorter than 2/3 of fore wing
40. Hamulohalterae: 0 – absent; 1 – present

Abdomen:

41. Abdomen connection to thorax: 0 – wide; 1 – narrow, stalk-like
42. Hypandrium (subgenital plate): 0 – absent; 1 – present

4. Phylogenetic analysis

The Maximum Parsimony (MP) analyses were performed in TNT 1.5[79,80], using the Traditional Search option, with memory reserved to store 99999 trees, 1000 replications, with 10 trees to save per replication; utilizing tree-bisection-reconnection (TBR) algorithm and collapsing zero-length branches, and Fulgoromorpha designated as the most distantly related outgroup taxon. The characters were treated as non-additive and unordered. We used two search strategies, including equal weights and implied weights[79,81]. For implied weights analyses, we tested a set of concavity (k) values from 1 to 11 and found no changes to the tree topology. Branch support values were estimated using 10000 bootstrap replicates. Character mapping was done in WinClada v. 1.00.08 with Unambiguous Changes, Fast Optimization (ACCTRAN) and Slow Optimization (DELTRAN) options[82,83].

Bayesian Inference (BI) was conducted in MrBayes v. 3.2.6[84] running on CIPRES Science Gateway v. 3.3. (phylo.org). The data were analysed using the Mkv model[85] and default set-

tings for priors. All analyses used four chains (one cold and three heated) and two runs of 10000000 generations. The analyses were conducted using a gamma distribution. Convergence of the two runs was visualized in Tracer v1.6[86], and by examining potential scale reduction factor (PSRF) values and the average standard deviation of split frequencies in the MrBayes output. In Bayesian Inference analysis nodes with (BI) posterior probability (PP) > 0.80 are considered as well supported, nodes with PP = 0.70–0.80 weakly supported, and nodes with PP < 0.70 were considered to be unsupported.

Results

The Bayesian Inference (BI) analysis reached convergence with an average standard deviation of split frequencies well below 0.01 after 10000000 generations. The Maximum Parsimony (MP) analyses under equal and implied weights resulted in one most parsimonious tree (L = 55, CI = 0.74, RI = 0.76). Both phylogenetic methods were highly congruent in their resultant topologies, and MP and BI trees were topologically very close (Supplement 1 Figs S1, S2a-c).

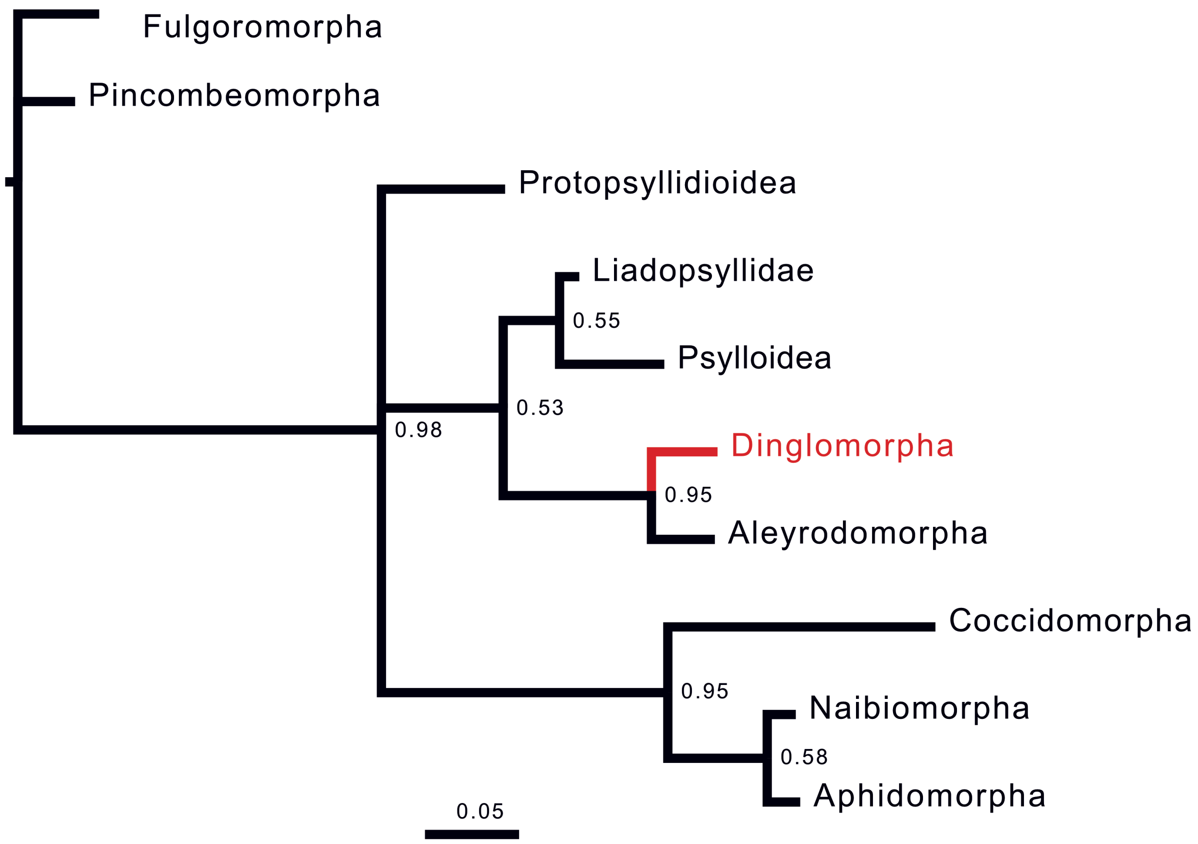

Fig. S1. Bayesian Inference tree of the Sternorrhyncha based on morphological dataset. Support values on branches are Bayesian posterior probability.

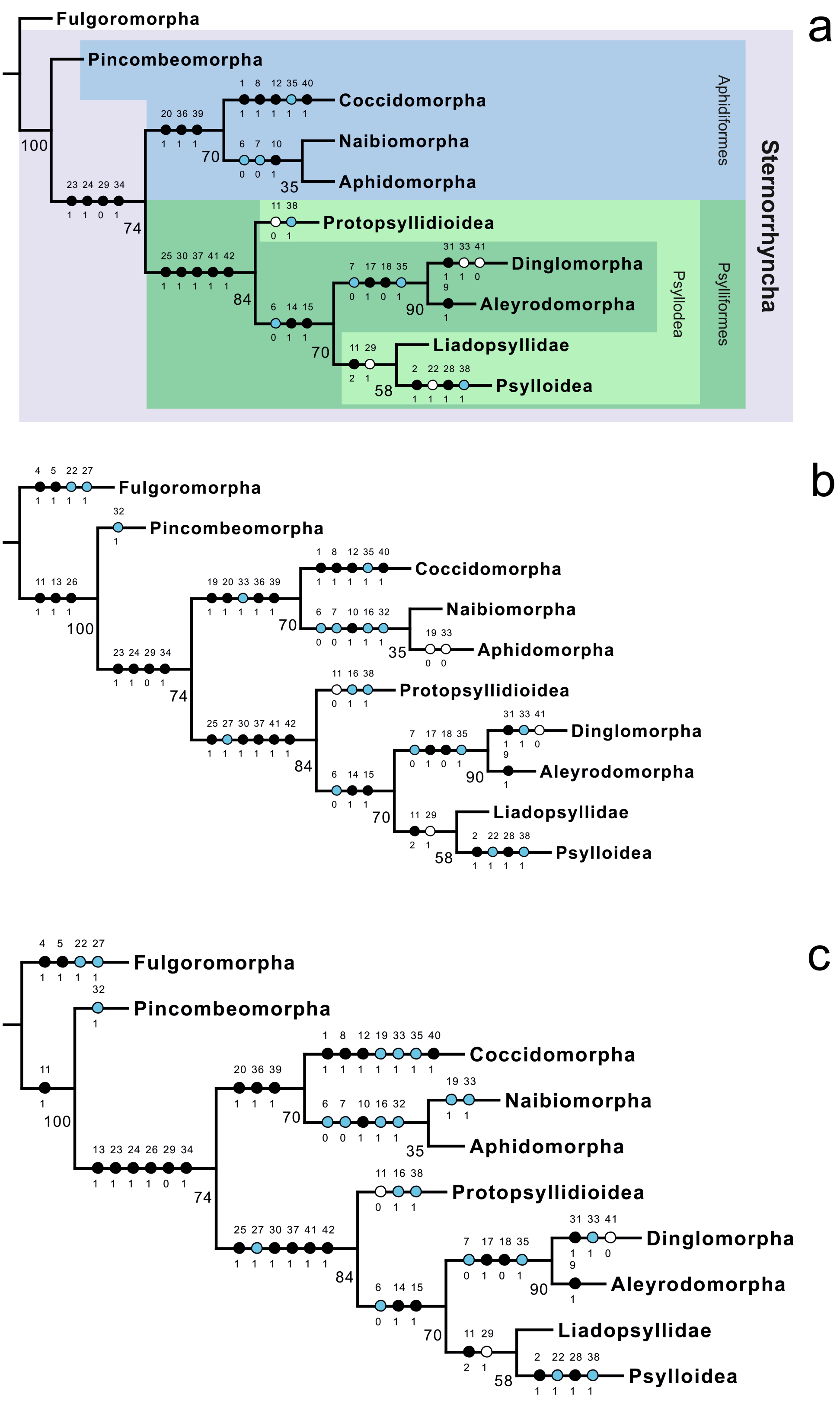

Fig. S2. Parsimony trees generated with TNT. Values at nodes indicate bootstrap supports. Unambiguous Changes Only (**a**), Fast Optimization (**b**), Slow Optimization (**c**). Black circles denote apomorphies, white – plesiomorphies, blue – homoplasies. Bootstrap values denoted at nodes. Colour frames denote the classification units.

The Sternorrhyncha, except for the extinct Pincombeomorpha, were recovered as monophyletic with strong support in the BI analysis (PP = 0.97), and relatively strong support in MP analyses (bootstrap value BS = 74). The first clade that branched off consists of Coccidomorpha that are resolved as sister (PP = 0.85; BS = 70) to Naibiomorpha + Aphidomorpha (no BI support, weakly supported (BS = 35). In the second clade, (with BI value of PP = 0.98; BS = 84), Protopsyllidioidea are recovered as sister to all remaining groups with strong support (PP = 0.91; BS = 70). Liadopsyllidae and Psylloidea were resolved as sister to each other, but without support in BI analysis, and weakly supported in MP analysis (BS = 58). The position of Dinglomorpha was recovered as sister to Aleyrodomorpha, well supported (PP = 0.95; BS = 90) and both as sister to Liadopsyllidae + Psylloidea (no support).

The results of the analyses of characters of all Sternorrhyncha, including the infraorders known only from the fossils, are presented below. The new infraorder Dinglomorpha **infraord. nov.** was resolved as sister group to Aleyrodomorpha, supported by two synapomorphies: reduced, membranous mesoscutellum [character 17(1)], and well developed metapostnotum [18(0)]. Presence of antennal flagellum [7(0)] appeared as a homoplastic feature, present also in the Naibiomorpha + Aphidomorpha clade. The absence of the areola postica [35(1)] is homoplastic and most likely related to the reduction of size and venation. Liadopsyllidae + Psylloidea clade (Psyllodea Flor, 1861) is supported by a single synapomorphy, the rostrum fused with prothoracic sternite [11(2)], and was revealed as sister to the Dinglomorpha + Aleyrodomorpha clade. Both clades are united by two synapomorphies, the mesoscutum and mesopraescutum comparable in size [14(1)] and mesopraescutum projected anteriad of the mesoscutum [15(1)]. Protopsyllidioidea is a lineage defined by homoplasies and a single plesiomorphy, free rostrum, not adpressed to sternum [11(0)]. According to the results of the MP analysis, Protopsyllidioidea are sister to [[Dinglomorpha + Aleyrodomorpha] + [Liadopsyllidae + Psylloidea]] clade, and the whole lineage is supported by number of synapomorphies, vein Pc not carinate [25(1)], vein ScP, short, not reaching margin [30(1)], thickened ambient vein present [37(1)], abdomen narrowly connected to thorax [41(1)] and hypandrium present [42(1)].

The extinct infraorder Naibiomorpha appears sister to Aphidomorpha, the clade is supported by a single synapomorphy, which is ‘the imaginal compound eye with additional ocellar structure’ [10(1)]. Coccidomorpha is sister group to Naibiomorpha + Aphidomorpha clade, the relationship is supported by several synapomorphies: ‘bases of legs on side of body’ [20(1)], ‘clavus reduced to absent’ [36(1)] and ‘hind wings reduced in size’ [39(1)]. The [19(1)], ‘metathoracic tergite in form of vestigial sclerite’ character was revealed as a synap-

omorphy for the Aphidiformes clade (Aphidococca), viz. [Coccidomorpha + [Naibiomorpha + Aphidomorpha]] in Fast Optimization procedure, with reversion of this state in Aphidomorpha.

This whole lineage is supported by four synapomorphies: ‘tarsi one or two segmented’ [23(1)], basal cell absent [24(1)], ‘costal cell between PC+CA+CP and ScP+R narrow’ [29(0)] and ‘crossvein rp-mp absent’ [34(1)]. The entire Sternorrhyncha clade is supported by several synapomorphies: ‘rostrum placed in sternal depression’ [11(1)], ‘mesonotum strongly raised, mesoscutum forming two humps’ [13(1)] and ‘veinlet cua-cup at basal cell absent’ [26(1)].

5. Systematic palaeontology
(with detailed descriptions)

Class Insecta Linnaeus, 1758[87]

Order Hemiptera Linnaeus, 1758[87]

Suborder Sternorrhyncha Amyot et Audinet-Serville, 1843[88]

Clade Psylliformes *sensu* Schlee, 1969[36] (= Psyllaleyroda *sensu* Kluge, 2010)[89]

Remark. This clade was proposed by Schlee[36] for the lineage uniting psyllids and whiteflies, including the extinct groups. Kluge[89] proposed a new taxon (clade) uniting modern psyllids and whiteflies in his circumscriptional nomenclature and classification system.

Dinglomorpha Szwedo & Drohojowska infraord. nov.

Diagnosis. Fore wing with costal veins complex carinate (Pc carinate as in all Psylliformes), ScP present as separate fold at base (unique feature) of common stem R+MP+CuA; common as in Aleyrodoidea); clavus present, with single claval vein A_1. Hypandrium present as small plate (as in Psylliformes).

Dingloidea Szwedo & Drohojowska superfam. nov.

Diagnosis. Fore wing membranous with modified venation – veins thickened, areola postica reduced; antennae 10-segmented; 3 ocelli present; stem MP present, connected with RP and CuA; abdomen widely fused with thorax; no wax glands on sternites.

Dinglidae Szwedo & Drohojowska fam. nov.

Type genus. Dingla Szwedo et Drohojowska **gen. nov.**; by present designation.

LSID urn:lsid:zoobank.org:act:D0A1C785-62D3-4E07-9A3B-FFAE3C13B704

Diagnosis. Imago. Head with compound eyes narrower than thorax. Eyes entirely rounded, postocular tumosity present; lateral ocelli placed dorsolaterally, near anterior angle of compound eye in dorsal view, median ocellus present. Antennae 10-segmented, with bases in frons to compound eyes, rhinaria scarce (?). Pronotum in mid line longer than mesopraescutum. Fore wing with thickened costal margin, basal portion of stem R+MP+CuA weak, distal portion of stem R+MP+CuA convex, forked at about half of fore wing length, branch RA short; pterostigmal area thickened. Common stem MP+CuA short, branches RP, MP and CuA parallel on membrane. Rostrum reaching metacoxae. Metacoxa without meracanthus. Metadistitarsomere longer than metabasitarsomere, claws distinct, long and narrow, no distinct additional tarsal structures. Male anal tube long. Hypandrium in form of small plate, styli long, narrow and acutely hooked at apex.

***Dingla* Szwedo & Drohojowska gen. nov.**

Type species. Dingla shagria Szwedo et Drohojowska **sp. nov.**; by present designation and monotypy.

LSID urn:lsid:zoobank.org:act:5053D386-4A13-445C-8036-9C69D885561F

Etymology. The generic name is derived from the adjective ‘dingla’ meaning ‘old’ in Jingpho language, which is spoken in Kachin state where the amber originates from. Gender: feminine.

Diagnosis. Vertex in mid line about as long as wide between compound eyes. Frons flat, widely triangularly incised at base. Antenna with 10th antennomere longer than penultimate one, widened, membranous apically, with terminal concavity. Pronotum about twice as wide as long. Mesopraescutum narrow, about as wide as pronotum; mesoscutum wide, with scutellar sutures not reaching anterior margin; mesoscutellum widely pentagonal. Fore wing with branch R forked anteriad of branch MP+CuA forking. Tip of clavus at level of MP+CuA forking. Hind wing with terminals RP and M subparallel and weakened in apical portion. Metafemur not thickened, metatibia without apical spines.

***Dingla shagria* Szwedo & Drohojowska sp. nov.**

LSID urn:lsid:zoobank.org:act:3EA05FB0-B783-4D7A-98EA-10B02F50B83D

Figs 2, 3, Supplement Fig. S3a-h

Etymology. The specific epithet is derived from the noun 'shagri' meaning 'insect' in Jingpho language spoken in the Kachin State, when the amber was collected.

Material. Holotype male. MAIG 5979, IAA FT-IR examination certificate 9791, registered as MAIG 5979IR (Supplement 1 Fig. S3a, b), Paratype male, MAIG 5980, IAA FT-IR examination certificate 9794, registered as MAIG 5980IR (Supplement 1 Fig. S3c, d), deposited in Museum of Amber Inclusions, Laboratory of Evolutionary Entomology and Museum of amber Inclusions, Department of Invertebrate Zoology and Parasitology, Faculty of Biology, University of Gdańsk, Gdańsk, Poland; paratype male NIGP172398 IAA FT-IR examination certificate IAA9792 (Supplement 1 Fig. S3e, f), paratype male NIGP172398, IAA FT-IR certificate IAA9793 (Supplement 1 Fig. S3g, h) deposited in Nanjing Institute of Geology and Palaeontology, Chinese Academy of Sciences, Nanjing, China.

Diagnosis. Pedicell, 2nd antennomere elongate, slightly thickened, 3rd antennomere longer than second and 4th; antennomeres 4th to 8th subequal in length. Protibia with row of thin setae in apicad half. Probasitarsomere about half as long as prodistitarsomere. Subgenital plate small, subquadrate, parameres long and narrow, parallel; about 3 times as long as wide at base, with hooked acute apex. Male anal tube tubular, slightly widening apicad, merely shorter than parameres.

Description. Male. Measurements (in mm): Total length 1.76 to 2.13; Body length total (including claspers) 1.76–2.13. Head including compound eyes width 0.37–0.52; head length along mid line 0.18–0.24; vertex width 0.2–0.26. Forewing length 1.32–1.79; forewing width 0.62–0.74; Claspers length 0.2–032. Antennomere 1st 0.04–0.08; antennomere 2nd 0.8–0.13; antennomere 3rd 0.08–0.16; antennomere 4th 0.06–0.12; antennomere 5th 0.06–0.09; antennomere 6th 0.06–0.1; antennomere 7th 0.06–0.09; antennomere 8th 0.0–0.09; antennomere 9th 0.06–0.09; antennomere 10th 0.0.8–0.01. Profemur+protrochanter cumulative length 0.26–0.46; protibia length 0.29–0.34; probasitarsomere length 0.06–0.09; prodistitarsomere length 0.08–0.13; mesofemur+mesotrochanter cumulative length 0.3–0.4; mesotibia length 0.36–0.4; mesobasitarsomere length 0.05–0.1; mesodistitarsomere length 0.13–015; metafemur+metatrochanter cumulative length 0.39–0.56; metatibia length 0.5–0.68; metabasitarsomere length 0.1–0.15; metadistitarsomere length 0.1–0.18.

Vertex about half as long as width of head with compound eyes; slightly narrower than wide at base; disc of vertex slightly concave; sutura coronalis absent. Scapus cyllindrical, longer than wide, pedicel slightly longer than scapus, barrel-shaped, wider than 3rd antennomere. Antennomere 3rd longer than 2nd antennomere (pedicel) antennomeres 5th to 9th subequal in length; antennomere 9th with subapical rhinarium; antennomere 10th (apical) longer then penultimate one, spoon-like widened apically, with rhinarium placed subapically. Median and lateral ocelli visible from above. Compound eyes large, not divided, with distinct, non-differentiated ommatidia; postocular protuberances narrow. Frons convex, with distinct triangular, concave median portion; median ocellus at margin with vertex; postclypeus and apical portion of loral plates distinctly incised to frons; postclypeus about twice as long as wide; anteclypeus tapering ventrad; lora semicircular, long, with upper angles slightly below upper margin of postclypeus, lower angles not exceeding half of anteclypeus length. Rostrum with apex reaching metacoxae; scapus short, wide, placed in distinct anterolateral concavity.

Pronotum massive, as long lateral as in midline; about 2.6 times as wide as long in mid line; disc of pronotum convex; anterior margin convex, slightly protruding between compound eyes; posterior margins converging posteriad; posterior margin slightly concave. Mesopraescutum with anterior margin covered by pronotum, with anterior margin convex, lateral margins expanded posterolaterad, with posterior margin convex posteriomediad, slightly concave posterolaterad. Mesoscutum distinctly wider than long in mid line; anterior margin merely concave medially, lateral margins distinctly diverging posteriad, posterolateral angles acute, distinct, posterior margin W-shaped, with distinct median concavity; disc of mesoscutum convex with indistinct longitudinal concavities (apodemes? sutures?). Mesoscutellum narrow, with anterior margin acutely convex, lateral margins subparallel, posterior margin straight, disc of mesoscutellum concave, with posteromedian furrow. Metascutum and metascutellum not visible.

Fore wing about 2.5 times as long as wide; narrower at base, widening posteriad, rounded in apical margin; widest at ¾ of its length. Costal margin thickened, veins thick, distinctly elevated; basal portion of stem R+MP+CuA weak, distal portion of stem R+MP+CuA convex, forked at about half of forewing length, branch RA short; pterostigmal area thickened; common stem MP+CuA short, branches RP, MP and CuA parallel on membrane; areola postica absent; clavus present, with apex exceeding half of forewing, with single claval vein A_1.

Hind wing about 0.8 times as long as forewing, with costal margin with two groups of regularly dispersed setae, basal group with seven longer and stiff setae and median group with 10 shorter, stout setae; terminals RP and M subparallel and weakened in apical portion.

Profemur and mesofemur subequal in length; protibia slightly shorter than mesotibia; pro- and metadistitarsomeres slightly longer than pro- and mesobasitarsomeres. Metacoxa without meracanthus; metafemur longer than pro- and mesofemur; metatibia distinctly longer than pro- and mesotibia; metadistitarsomere distinctly longer than metabasitarsomere; tarsal claws long, narrow, without arolium or empodium.

Abdomen with segments III to VIII almost homonomic in length, widely connected to thorax, subgenital portion narrowing. Subgenital plate small, subquadrate, parameres long and narrow, parallel; about 3 times as long as wide at base, with hooked acute apex. Male anal tube tubular, slightly widening apicad, merely shorter than parameres.

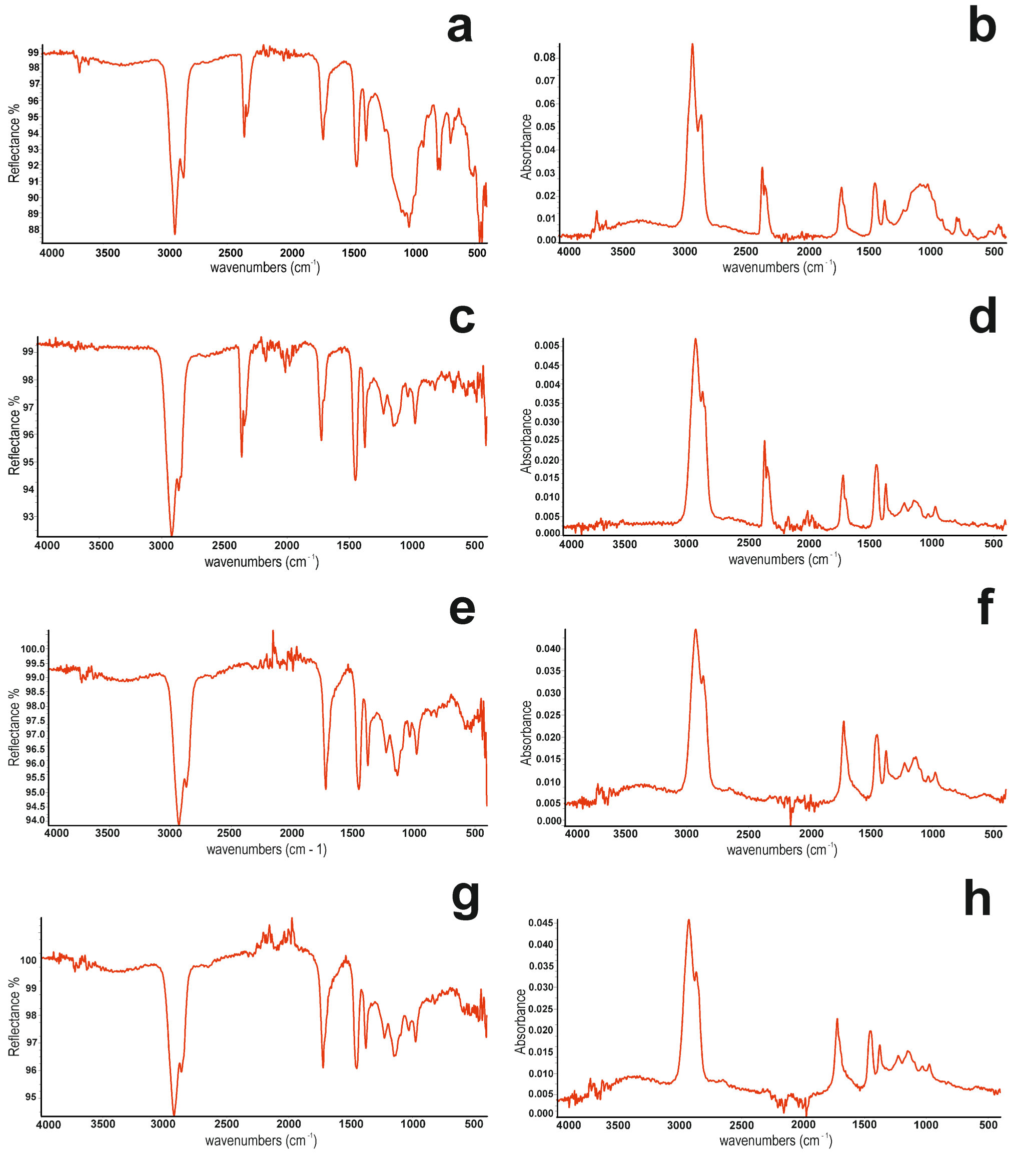

Fig. 3. FT-IR spectra of analysed amber pieces. Holotype MAIG 5979: reflectance spectrum (**a**), ATR corrected absorbance spectrum (**b**); Paratype MAIG 5980: reflectance spectrum (**c**), ATR corrected absorbance spectrum (**d**); Paratype NIGP172398: reflectance spectrum (**e**), ATR corrected absorbance spectrum (**f**); Paratype NIGP172399: reflectance spectrum (**g**), ATR corrected absorbance spectrum (**h**).

6. Fossil record, classification and phylogeny of Sternorrhyncha

The modern treatment of the Sternorrhyncha with two distinguished clades (Supplement Fig. S2a) results from the data and their interpretations presented by Schlee[36], Shcherbakov[26], Kluge[28,89] and Gavrilov-Zimin *et al.*[90]. Within the suborder Sternorrhyncha, two clades – the Aphidiformes (= Aphidococca) covering Pincombeomorpha, Aphidomorpha, Naibiomorpha and Coccidomorpha and Psylliformes (= Psyllaleyroda) with Aleyrodomorpha and Psyllodea[1]. The summary of names and content of taxa mentioned is given below (Supplement Table 3).

Table S3. Sternorrhyncha classification

Subordo Sternorrhyncha Amyot et Audinet Serville, 1843[88]

Cladus Aphidiformes *sensu* Schlee, 1969[36] (=Aphidococca *sensu* Kluge, 2010)[28]

Infraordo Pincombeomorpha Shcherbakov, 1990[91]

Infraordo Coccidomorpha Heslop-Harrison, 1952[92]

Infraordo Naibiomorpha Szwedo, 2018[1]

Infraordo Aphidomorpha Becker-Migdisova et Aizenberg, 1962[93]

Cladus Psylliformes *sensu* Schlee, 1969[36] (= Psyllaleyroda *sensu* Kluge, 2010)[28]

Infraordo Aleyrodomorpha Chou, 1963[94]

Infraordo Dinglomorpha Szwedo et Drohojowska infraord. nov.

Infraordo Psyllodea Flor, 1861[95] (=Psyllaeformia Verhoeff, 1893)[96]

Superfamilia Protopsyllidioidea Carpenter, 1931[a,97]

Superfamily Psylloidea Latreille, 1807

[a] most probably paraphyletic assemblage

Reconstructing the phylogenetic relationships of modern Sternorrhyncha based on molecular data is not an easy task. Firstly, the selection of taxa already analysed is not comprehensive and data for numerous subunits and groups are not available. Secondly, the problem in sternorrhynchan molecular phylogenetics, which was very well addressed rather early on, is the long-branch attraction phenomenon. It was pointed out that psyllids show deviations from

the normal 18S rDNA sequence in places of insertions characteristic of the remaining Sternorrhyncha, hinting that these insertions had once been present, but later became lost[99], and that long-branch attraction may explain association of unusually long 18S rDNA sequences of whiteflies and aphids. Analysis of the whole mitochondrial genomes of whiteflies, aphids and psyllids[100] revealed that gene sequences in aphids and psyllids are conservative, while in whiteflies there were variations in mitochondrial gene order. Other phylogenetic studies of Hemiptera[101-104] have shown that the aphids should be one of the closest relatives of whiteflies. Both of the groups have similar biologies, viz. faster generation times and more generation, yet the aphids' mitogenomes exhibit lower sequence evolutionary rate and shorter branch lengths than whiteflies[104-106]. Very little attention was paid to including and explaining mitochondrial genome-based topologies with the addition of scale insects to psyllids, whiteflies and aphids. According to the available results of the whole mitogenomes analysis[107], aphids and coccids form a clade, sister to whiteflies, and together sister to psyllids. The same relationship pattern was revealed in the analyses of amino acid transporter genes[108] and increased mitogenome sequences[109]. The alternative relationship hypothesis with aphids and coccids as a clade, sister to psyllids and whiteflies as sister to remaining sternorrhynchans was presented by Johnson *et al.*[34] and Wang Y.H. *et al.*[110], both based on transcriptomes (Supplement Fig. S4C). Similar results, where whiteflies were resolved outside of other Sternorrhyncha (no coccids included in the analyses) were obtained by Song *et al.*[104] based on mitogenomes, and Song *et al.*[111] combining mitogenomes and nuclear genes.

The oldest Aphidomorpha (Supplement Fig. S5a, c) are known from the Artinskian (early Permian) of Lodève, France[30], the Coccidomorpha (Supplement Fig. S5a, f) enter the fossil record very late and their fossils are known since the Valanginian (early Cretaceous) of the United Kingdom[111], however a much older origin of the group, at least Triassic, was postulated by[112]. The host(s), habitat, life behaviour and early phylogeny stages of Coccidomorpha remained hidden until the evolution of flowering plants had begun when coccids "suddenly" appeared in numerous and diverse groups[1,114,115]. The dearth of pre-Cretaceous fossils may be due to palaeoecological or taphonomical conditions (litter and underground life style of early coccidomorphs hypothesized by Koteja[113]), as well as failure to recognize early scale insect fossils when sorting the material. The extinct Naibiomorpha (Supplement Fig. S5a, d) are known since the Ladinian (middle Triassic) of Tongchuan Formation, Shaanxi Province, China[116]. Another extinct lineage – the Pincombeomorpha (Supplement Fig. S5a, c) enter the fossil record in the Kungurian (terminal early Permian) of Koshelevka Formation, Tshekarda, Ural Mountains, Russian Federation[9]. The oldest Psylliformes, viz. unidentified Protopsyllid-

ioidea are reported from Kungurian (lower Permian), carbonaceous shales of middle Ecca Group, Haakdoornfontein, South Africa (Supplement Fig. 5a, c)[117]; the oldest modern Psylloidea s.l. – Liadopsyllidae (Supplement Fig. 5a, e)[48], appeared in the Toarcian of Grimmen, Germany[118]. Modern Psylloidea s. str. are known as fossils since Lutetian of Kishenehn Formation, Montana, U.S.A. and Baltic amber (Supplement Fig. 5a, g)[1,119]. The oldest Aleyrodoidea appeared in the Callovian/Oxfordian (middle/late Jurassic) of Daohugou, China (Supplement Fig. 5a, e)[27]. The record of particular lineages and their diversification, palaeodiversity and palaeo disparity is very uneven, but at least some general evolutionary patterns

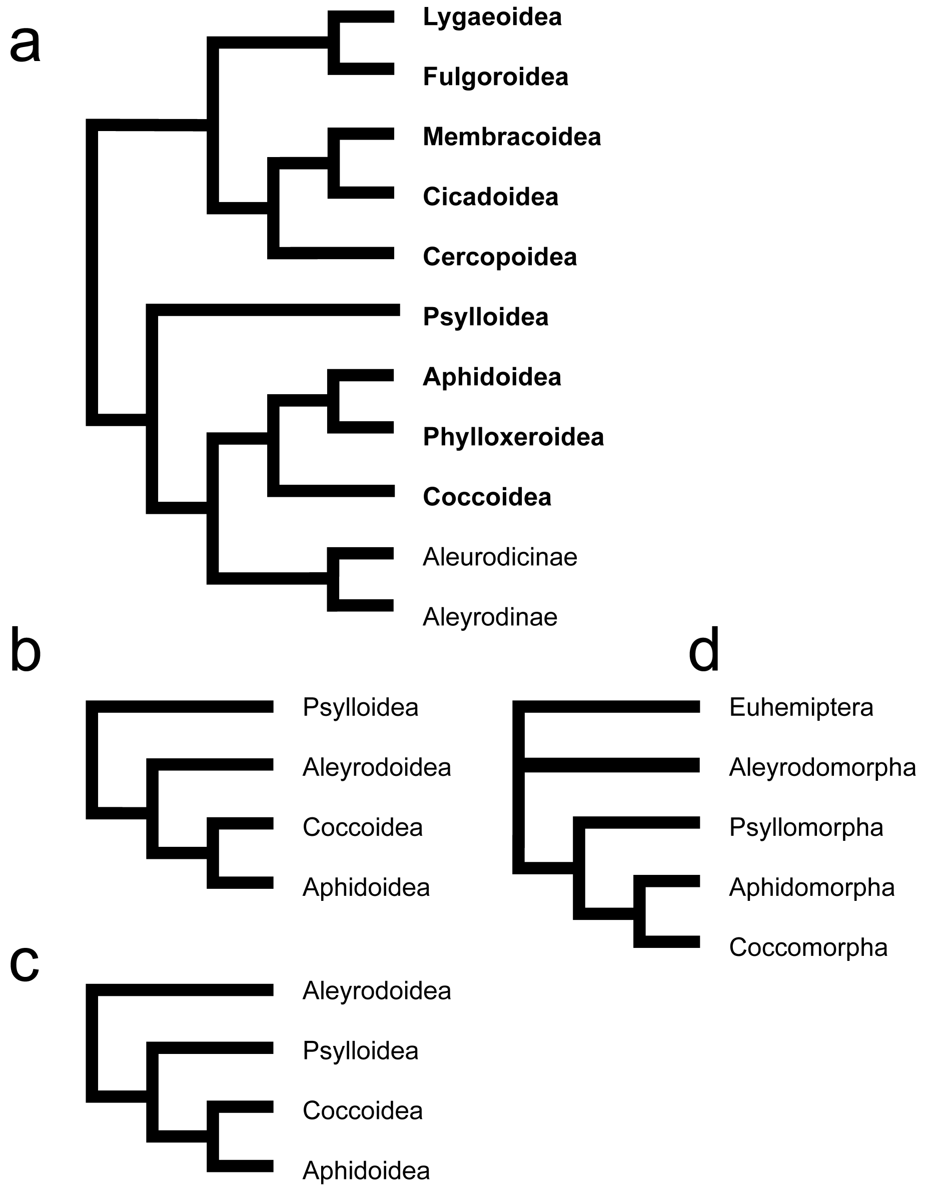

Fig. S4a-d. Relationships within Sternorrhyncha according to various authors. According to Campbell *et al.*[133] (**a**), according to Song *et al.*[108] (**b**), according to Wang Y.H. *et al.*[110] (**c**), according to Wegierek[32] (**d**).

can be proposed (Supplement Fig. S5). The initial diversification of the earliest sternorrhynchans could be related to the development of secondary phloem in the lignophytes – seed plants and progymnosperms[120,121] in the Carboniferous. Findings of the sternorrhynchan nymphs, galls and feeding traces on Permian plants[122,123], together with taxonomic diversity, morphological specialization and disparity observed in the fossil record, suggest that phloem-feeding insects were important components of early ecosystems[124]. The Aphidiformes largely diversified in the Triassic and Jurassic with several lineages of aphids[125,126], naibiomorphans and pincombeomorphans[26], but as mentioned before, there is no fossil record of coccidiomorphan from these times (Supplement Fig. S5). This could be explained by taphonomic reasons, such as decay and scavenging and low preservation potential of diminutive forms[127,128]. Several morpho-ecological changes were proposed as a result of coccidomomorpha ancestors shifting from above ground habitats on plant stems, branches and twigs, as is still present in most of aphids, to hypogeic habitats, viz. litter and soil; as retained e.g. in the most basal ensign scale insects (Orthezioidea). These include the appearance of wingless, neotenic females, with marsupium; miniaturized, dipterous males; legs adapted for digging, not climbing[113].

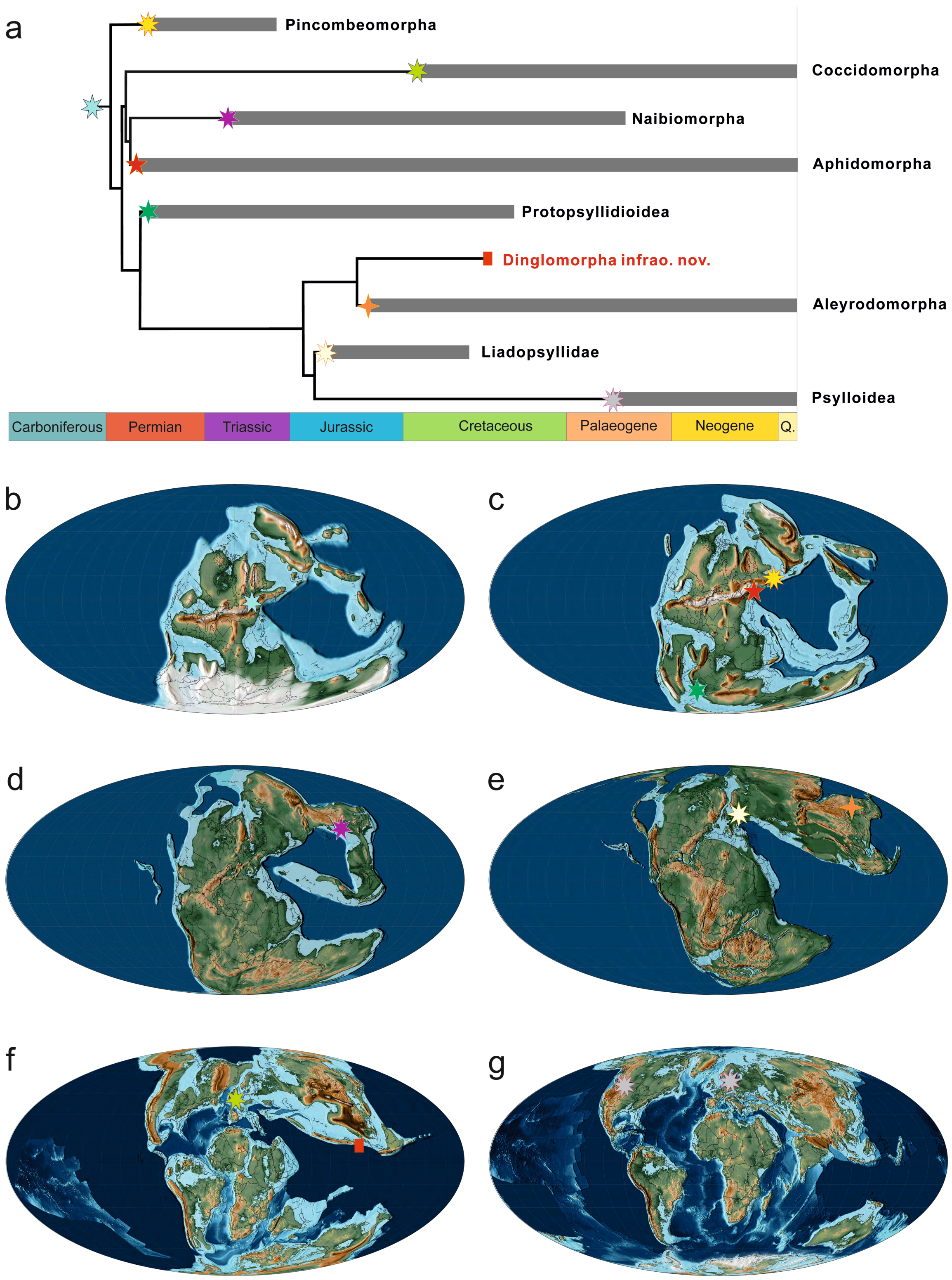

Fig. S5. Relationships and distribution of oldest Sternorrhyncha. Chronophyletic scheme of sternorrhynchan lineages (**a**); oldest record of Stenorrhyncha[5], palaeoglobe Moscovian (**b**); oldest records of Aphidomorpha[30], Pincombeomorpha[9], Protopsyllidioidea[117], palaeoglobe Artinskian; (**c**) oldest records of Naibiomorpha[116], palaeoglobe Anisian (**d**); oldest records of Liadopsyllidae[119] and Aleyrodomorpha[27], palaeoglobe Toarcian (**e**); oldest record of Coccidomorpha[112] and Dinglomorpha, palaeoglobe Albian (**f**); oldest records of Psylloidea s. str.[119], palaeoglobe Lutetian (**g**); palaeoglobes after Scotese[130-134].

Additional references

(not listed in the main text)

49. Kania, I., Wang, B. & Szwedo, J. *Dicranoptycha* Osten Sacken, 1860 (Diptera, Limoniidae) from the earliest Cenomanian Burmese amber. *Cret. Res.*, **52**, 522–530; 10.16/j.cretres.2014.03.002 (2015).
50. Thu, K. & Zaw, K. Gem deposits of Myanmar in *Myanmar Geology*, *Resources and Tectonics* (eds Barber, A. J., Zaw, K. & Crow, M. J.) *Mem. Geol. Soc. London* **48**, 497–529; 10.1144/M48.23 (2017).
51. Grimaldi, D.A., Engel, M.S. & Nascimbene, P.C. Fossiliferous Cretaceous amber from Myanmar (Burma): its rediscovery, biotic diversity, and paleontological significance. *Am. Mus. Novit.* **3361**, 1–71; 10.1206/0003-00823612.0.CO;2 (2003).
52. Cruickshank, R.D. & Ko, K. Geology of an amber locality in the Hukawng Valley, Northern Myanmar. *J. Asian Earth Sci.* **21**, 441–455; 10.1016/S1367-9120(02)00044-5 (2003).
53. Shi, G.H. *et al.* Age constraint on Burmese amber based on U-Pb dating of zircons. *Cret. Res.* **37**, 155–163; 10.1016/j.cretres.2012.03.014 (2012).
54. Zheng, D. *et al.* A well-preserved true dragonfly (Anisoptera: Gomphides: Burmagomphidae fam. nov.) from Cretaceous Burmese amber. *J. Syst. Palaeontol.* **16** (10), 881–889; 10.1080/14772019.2017.1365100 (2018).
55. Grimaldi, D.A. & Ross, A.J. Extraordinary Lagerstätten in amber, with particular reference to Cretaceous of Burma in *Terrestrial conservation Lagerstätten: windows into the evolution of life on land* (ed. Fraser, N. & Sues, H.-D.) 287–342 (Dunedin Academic Press, Edinburgh 2017).
56. Smith, R.D.A. & Ross, A.J. Amberground pholadid bivalve borings and inclusions in Burmese amber: implications for proximity of resin-producing forests to brackish waters, and the age of the amber. *Earth Env. Sci. T.R. So.* **107** (2-3), 239–247; 10.1017/S1755691017000287 (2018).
57. Yu, T.T. *et al*. An ammonite trapped in Burmese amber. *Proc. Natl. Acad. Sci. USA* **116** (23), 11345–11350; 10.1073/pnas.1821292116 (2019).
58. Helm, O. On a new, fossil, amber-like resin occurring in Burma. *Rec. Geol. Surv. Ind.* **25** (4), 180–181 (1892).
59. Helm, O. Further note on Burmite, a new amber-like fossil resin from Upper Burma. *Rec. Geol. Surv. Ind.* **26** (2), 61–64 (1893).

60. Noetling, F. On the occurrence of Burmite, a new fossil resin from Upper Burma. *Rec. Geol. Surv. Ind.* **26** (2), 31–40 (1893).
61. Poinar, G. Jr., Lambert, J.B. & Wu, Y. Araucarian source of fossiliferous Burmese amber: spectroscopic and anatomical evidence. *J. Bot. Res. Inst. Texas* **1**, 449–455 (2007).
62. Dutta, S., Mallick, M., Kumar, K., Mann, U. & Greenwood, P.F. Terpenoid composition and botanical affinity of Cretaceous resins from India and Myanmar. *Int. J. Coal Geol.* **85** (1), 49–55; 10.1016/j.coal.2010.09.006 (2011).
63. Metcalfe, I. Gondwana dispersion and Asian accretion: Tectonic and palaeogeographic evolution of eastern Tethys. *J. Asian Earth Sci.* **66**, 1–33; 10.1016 /j.jseaes.2012.12.020 (2013).
64. Cleal, C. & Thomas, B. *Introduction to plant fossils*. 2nd edition. x+1–254 (Cambridge University Press, Cambridge, UK 2019); 10.1017/9781108650021.
65. Xing, L. *et al.* A gigantic marine ostracod (Crustacea: Myodocopa) trapped in mid-Cretaceous Burmese amber. *Sci. Rep.* **8** (1365), 1–9; 10.1038/s41598-018-19877-y (2018a).
66. Mao, Y. *et al.* Various amber ground marine animals in Burmese amber with discussions on its age. *Palaeoentomology* **1** (1), 91–103; 10.11646/palaeoentomology.1.1.11 (2018).
67. Broly, P., Maillet, S., & Ross, A.J. The first terrestrial isopod (Crustacea: Isopoda: Oniscidea) from Cretaceous Burmese amber of Myanmar. *Cret. Res.* **55**, 220–228; 10.1016/j.cretres.2015.02.012 (2015).
68. Westerweel, J. *et al.* Burma Terrane part of the Trans-Tethyan arc during collision with India according to palaeomagnetic data. *Nat. Geosci.* **12**, 863–868; 10.1038/s41561-019-0443-2 (2019).
69. Heine, C. & Müller, R. Late Jurassic rifting along the Australian North West Shelf: margin geometry and spreading ridge configuration. *Austral. J. Earth Sci.* **52**, 27–39; 10.1080/08120090500100077 (2005).
70. Seton, M. *et al.* Global continental and ocean basin reconstructions since 200Ma. *Earth-Sci. Rev.* **113**, 212–270; 10.1016/j.earscirev.2012.03.002 (2012).
71. Licht, A. *et al.* Paleogene evolution of the Burmese forearc basin and implications for the history of India-Asia convergence. *Geol. Soc. Am. Bull.* **130** (5-6), 730–748; 10.1130/B35002.1 (2019).
72. Jiang, T., Szwedo, J. & Wang, B. A giant fossil Mimarachnidae planthopper from the mid-Cretaceous Burmese amber (Hemiptera, Fulgoromorpha). *Cret. Res.* **89**, 183–190; 10.1016/j.cretres.2018.03.020 (2018).

73. Rasnitsyn, A.P. & Öhm-Kühnle, C. Three new female *Aptenoperissus* from mid-Cretaceous Burmese amber (Hymenoptera, Stephanoidea, Aptenoperissidae): Unexpected diversity of paradoxical wasps suggests insular features of source biome. *Cret. Res.* **91**, 168–175; 10.1016/j.cretres.2018.06.004 (2018).
74. Koteja, J. Morphology and taxonomy of male Ortheziidae (Homoptera, Coccinea). *Pol. J. Entomol.* **56**, 323–374 (1986).
75. Franielczyk-Pietyra, B. & Wegierek, P. The forewing of *Aphis fabae* (Scopoli, 1763) (Hemiptera, Sternorrhyncha): a morphological and histological study. *Zoomorphology* **136** (3), 349–358; 10.1007/s00435-017-0358-7 (2017).
76. Franielczyk-Petyra, B., Depa, Ł. & Wegierek, P. Morphological and histological study of the forewing of *Aleyrodes proletella* (Linnaeus, 1758) (Sternorrhyncha, Hemiptera) with a comparative analysis of forewings among Sternorrhyncha infraorders. *Zoomorphology* **138** (3), 321–333; 10.1007/s00435-019-00449-1 (2019).
77. Nel, A., Prokop, J., Nel, P. & Grandcolas, P. Traits and evolution of wing venation pattern in Paraneopteran insects. *J. Morphol.* **273** (5), 480–506; 10.1002/jmor.11036 (2012).
78. Maddison, W.P. & Maddison, D.R. Mesquite: a modular system for evolutionary analysis. Version 3.61. http://www.mesquiteproject.org (2019).
79. Goloboff, P.A., Farris, J.S. & Nixon, K.C. TNT, a free program for phylogenetic analysis. *Cladistics* **24** (5), 774–786; 10.1111/j.1096-0031.2008.00217.x (2008).
80. Goloboff, P. A. & Catalano, S. A. TNT version 1.5, including a full implementation of phylogenetic morphometrics. *Cladistics* **32,** 221–238; 10.1111.cla.12160 (2016).
81. Congreve, C.R. & Lamsdell, J.C. Implied weighting and its utility in palaeontological datasets: a study using modelled phylogenetic matrices. *Palaeontology* **59**, 447–462; 10.1111/pala.12236 (2016).
82. Nixon, K.C. WinClada Ver. 1.00.08. Published by the Author, Ithaca; New York. http://www.cladistics.com/wincDownload.htm (2002).
83. Agnarsson, I. & Miller, J.A. Is ACCTRAN better than DELTRAN? *Cladistics*, **24** (6), 1032–1038; 10.1111/j.1096-0031.2008.00229.x (2008).
84. Ronquist, F. *et al.* MrBayes 3.2: efficient Bayesian phylogenetic inference and model choice across a large model space. *Syst. Biol.* **61,** 539–542; 10.1093/sysbio/sys029 (2012).
85. Lewis, P.O. A likelihood approach to estimating phylogeny from discrete morphological character data. *Syst. Biol.* **50** (6), 913–925 (2001).
86. Rambaut, A., Suchard, M., Xie, D. & Drummond, A. Tracer v1.6 http://beast.bio.ed.ac.uk/Tracer (2014).

87. Linnaeus, C. *Systema naturae per regna tria naturae, secundum classes, ordines, genera, species, cum characteribus, differentiis, synonymis, locis. Tomus I. Editio decima, reformata*. (Laurentii Salvii, Holmiæ 1758).
88. Amyot, C.J.-B. & Audinet-Serville, J.G. *Deuxième partie. Homoptères. Homoptera Latr. Histoire Naturelle des insectes. Hemiptères*, 1–676 (Librairie encyclopédique de Roret, Paris 1843).
89. Kluge, N.Yu. Paradoxical molting process in *Orthezia urticae* and other coccids (Arthroidignatha: Gallinsecta) with notes on systematic position of scale insects. *Zoosyst. Ross.* **19**, 246–271 (2010b).
90. Gavrilov-Zimin, I., Stekolshchikov, A. & Gautam, D.C. General trends of chromosomal evolution in Aphidococca (Insecta, Homoptera, Aphidinea + Coccinea). *Comp. Cytogenet.* **9** (3), 335–422; 10.3897/CompCytogen.v9i3.4930 (2015).
91. Shcherbakov, D.E. Extinct four-winged ancestors of scale insects (Homoptera: Sternorrhyncha) in *Proceedings of the Sixth International Symposium of scale insect Studies*, part II, Cracow, August 6–12 1990, (ed. Koteja, J.) 23–29 (Agricultural University Press, Kraków 1990).
92. Heslop-Harrison, G. LXXII. Preliminary notes on the ancestry, family relations, evolution and speciation of the Homopterous Psyllidae. II. *Ann. Mag. Nat. Hist.* Ser. 12, **5** (55), 679–696; 10.1080/00222935208654339 (1952).
93. Becker-Migdisova, E.E. & Aizenberg, E.E. Infraotryad Aphidomorpha. [Infraorder Aphidomorpha] in *Osnovy paleontologii. Chlenistonogie. Trakheı̆nye i khelitserovye* (ed. Rodendorf, B.B.) **9,** 194–199 (Akademia Nauk SSSR, Moskva1962). [Published in English as: Becker-Migdisova, E.E. & Aizenberg, E.E. Infraorder Aphidomorpha in *Principles of Palaeontology. Arthropoda. Tracheata and Chelicerata* (ed. Rohdendorf, B.B.) **9**, 267–274 (Smithsonian Institution Libraries and The National Science Foundation, Washington, DC 1991).
94. Chou, I. Some viewpoints about insect taxonomy. *Acta Entomol. Sinica*, **12**, 586–596 (1963).
95. Flor, G. Die Rhynchoten Livlands in systematische Folge beschrieben. Archiv für die Naturkunde Liv-, Ehst- und Kurlands 2, *Biol. Naturk.* **4**, 438–546 (1861).
96. Verhoeff, C.W. Vergleichende Untersuchungen über die Abdominalsegmente der weiblichen Hemiptera-Heteroptera und Homoptera, ein Beitrag zur Kenntniss der Phylogenie derselben. *Verhandl. d. naturh. Ver. d. Preuss. Rheinl. u. Westphal.* **50**, 307–374 (1893).

97. Carpenter, F.M. The Lower Permian insects of Kansas: Part 4. The order Hemiptera, and additions to the Paleodictyoptera and Protohymenoptera. *Am. J. Sci.* **5** (22), 113–30 (1931).
98. Latreille, P.A. *Sectio secunda. Familia quarta. Cicadariae. Cicadaires. In Genera crustaceorum et insectorum: secundum ordinem natrualem in familias disposita, iconibus exemplisque plurimis explicata* **3**, 1–258 (Amand Koenig Paris 1807).
99. Aleshin, V.V., Vladychenskaya, N.S., Kedrova, O.S., Milyutina, I.A. & Petrov, N.B. Phylogeny of invertebrates deduced from 18S rRNA comparisons. *Mol. Biol. (Mosk.)* **29** (6), 843–855 (1995).
100. Thao, M.L., Baumann, L. & Baumann, P. Organization of the mitochondrial genomes of whiteflies, aphids, and psyllids (Hemiptera, Sternorrhyncha). *BMC Evol. Biol.* **4**, 25, 1–13; 10.1186/1471-2148-4-25 (2004).
101. Campbell, B.C., Steffen-Campbell, J.D., Sorensen, J.T. & Gill, R.J. Paraphyly of Homoptera and Auchenorrhyncha inferred from 18S rRNA nucleotide sequences. *Syst. Entomol.* **20**, 175–194 (1995).
102. Dohlen, C.D. von & Moran, N.A. Molecular phylogeny of the Homoptera: a paraphyletic taxon. *J. Mol. Evol.* **41**, 211–223 (1995).
103. Cryan, J. & Urban, J. Higher-level phylogeny of the insect order Hemiptera: is Auchenorrhyncha really paraphyletic? *Syst. Entomol.* **37**, 7–21; 10.1111/j.1365-3113.2011.00611.x (2012).
104. Song, N., Liang, A.P. & Bu, C.P. A molecular phylogeny of Hemiptera inferred from mitochondrial genome sequences. *PLoS One* **7**, e48778, 1–13; 10.1371/journal.pone.0048778 (2012).
105. Cui, Y. *et al.* Phylogenomics of Hemiptera (Insecta Paraneoptera) based on mitochondrial genomes. *Syst. Entomol.* **38**, 233–245; 10.1111/j.1365-3113.2012.00660.x (2013).
106. Wang, Y., Huang, X.L. & Qiao, G.X. Comparative analysis of mitochondrial genomes of five aphid species (Hemiptera: Aphididae) and phylogenetic implications. *PLoS One* **17**, 8(10), e77511, 1–13; 10.1371/journal.pone.0077511 (2013).
107. Li, H., *et al.* Mitochondrial phylogenomics of Hemiptera reveals adaptive innovations driving the diversification of true bugs. *Proc. R. Soc.* B, **284** (1862), 20171223, 1–10; 10.1098/rspb.2017.1223 (2017).
108. Dahan, R.A., Duncan, R.P., Wilson, A.C.C. & Dávalos, L.M. Amino acid transporter expansions associated with the evolution of obligate endosymbiosis in sap-feeding insects (Hemiptera: Sternorrhyncha). *BMC Evol. Biol.* **15** (52), 1–11; 10.1186/s12862-015-0315-3 (2015).

109. Song, N., Zhang, H. & Zhao, T. Insights into the phylogeny of Hemiptera from increased mitogenomic taxon sampling. *Mol. Phylog. Evol.* **137**, 236–249; 10.1016/j.ympev.2019.05.009 (2019).
110. Wang, Y.H. *et al*. When did the ancestor of true bugs become stinky? Disentangling the phylogenomics of Hemiptera-Heteroptera. *Cladistics* **35**, 42–66; 10.1111/cla.12232 (2019).
111. Song, N. *et al*. Phylogenetic relationships of Hemiptera inferred from mitochondrial and nuclear genes. *Mitochondrial DNA* Part A, **27** (6), 4380–4389; 10.3109/19401736.2015.1089538 (2016).
112. Koteja, J. *Eomatsucoccus andrewi* sp. nov. (Hemiptera: Sternorrhyncha: Coccinea) from the Lower Cretaceous of southern England. *Cret. Res.* **20**, 863–866 (1999).
113. Koteja, J. Essay on the prehistory of the scale insects (Homoptera, Coccinea). *Ann. zool.* **38** (15), 461–503 (1985).
114. Hodgson, C.J. & Hardy, N.B. The phylogeny of the superfamily Coccoidea (Hemiptera: Sternorrhyncha) based on the morphology of extant and extinct macropterous males. *Syst. Entom.* **38**, 794–804; 10.1111/syen.12030 (2013).
115. Vea, I.M. & Grimaldi, D.A. Putting scales into evolutionary time: the divergence of major scale insect lineages (Hemiptera) predates the radiation of modern angiosperm hosts. *Sci. Rep.* **6**, 23487, 1–11; 10.1038/srep23487 (2016).
116. Hong, Y.C., Zhang, Z.J., Guo, X.R. & Heie, O.E. A new species representing the oldest aphid (Hemiptera, Aphidomorpha) from the Middle Triassic of China. *J. Paleontol.* **83** (5), 826–831 (2009).
117. Geertsema, H., van Dijk, D.E., & van den Heever, J.A. Palaeozoic insects of southern Africa: a review. *Palaeontol. afr.* **38**, 19–25 (2002).
118. Ansorge, J. Insekten aus dem oberen Lias von Grimmen (Vorpommern, Norddeutschland). *Neue Paläontol. Abhandl.* **2**, 1–132 (1996).
119. Ouvrard, D., Burckhardt, D. & Greenwalt, D. The oldest jumping plant-louse (Hemiptera: Sternorrhyncha) with comments on the classification and nomenclature of the Palaeogene Psylloidea. *Acta Mus. Morav.*, Sci. biol. **98** (2), 21–33 (2013).
120. Taylor, T.N., Taylor, E.L. & Krings, M. *Palaeobotany. The biology and evolution of fossil plants*. Second edition xxi+1–1320 (Academic Press, Burlington MA. 2009).
121. Decombeix, A.-L., Galtiere, J., & Meyer-Berthaud, B. Secondary phloem in early Carboniferous seed plants: anatomical diversity and evolutionary implications. *Int. J. Plant Sci.* **175** (8), 891–910; 10.1086/677650 (2014).

122. Labandeira, C.C., Wilf, P., Johnson, K.P. & Marsh, F. *Guide to insect (and other) damage types on compressed plant fossils. Version 3.0. [WWW document].* 1–25 (Smithsonian Institution, Washington, D.C 2007).

123. Schachat, S.R. & Labandeira, C.C. Evolution of complex behavior: the origin and initial diversification of foliar galling by Permian insects. *Sci. Nat.* **102**, 14, 1–8; 10.1007/s00114-015-1266-7 (2015).

124. Shcherbakov, D.E. O permskikh i triasovykh entomofaunakh v svyazi s biogeografieï i permo-triasovym krizisom. *Paleontol. Zh.* **1**, 15–32 (2008). Published in English: Shcherbakov, D.E. On Permian and Triassic insect faunas in relation to biogeography and the Permian–Triassic crisis. *Paleontol. J.* **42** (1), 15–31; 10.1007/s11492-008-1003-1 (2008).

125. Heie, O.E. & Wegierek, P. A classification of the Aphidomorpha (Hemiptera Sternorrhyncha) under consideration of the fossil taxa. *Redia*, **42**, 69–77 (2009a).

126. Heie, O.E. & Wegierek, P. Diagnoses of the higher taxa of Aphidomorpha (Hemiptera Sternorrhyncha). *Redia*, **42**, 261–269 (2009b).

127. Martin, R.E. *Taphonomy. A process approach*. Cambridge Paleobiology Series **4**. (Cambridge University Press, Cambridge, UK 1999).

128. Allison, P.A. & Bottjer, D.J. *Taphonomy. Process and bias through time*. Second edition (Springer, Dordrecht Heidelberg London New York 2011).

129. Campbell, B.C., Steffen-Campbell, J.O. & Gill,·R.J. Evolutionary origin of whiteflies (Hemiptera: 5ternorrhyncha: Aleyrodidae) inferred from 185 rDNA sequences. *Insect Mol. Biol.* **3** (2), 73–88 (1994).

130. Scotese, C.R. The PALEOMAP Project PaleoAtlas for ArcGIS, ver. 2, Vol. 1, Cenozoic plate tectonic, paleogeographic, and paleoclimatic reconstructions, Maps 1–15, (PALEOMAP Project, Evanston, IL. 2014a).

131. Scotese, C.R. Atlas of Early Cretaceous paleogeographic maps, PALEOMAP Atlas for ArcGIS, Vol. 2, The Cretaceous, Maps 23–31, Mollweide projection (PALEOMAP Project, Evanston, IL. 2014b).

132. Scotese, C.R. Atlas of Middle & Late Permian and Triassic paleogeographic maps, maps 43–48 from Vol. 3, PALEOMAP Atlas for ArcGIS (Jurassic and Triassic) and maps 49–52, Vol. 4, PALEOMAP PaleoAtlas for ArcGIS (Late Paleozoic), Mollweide projection (PALEOMAP Project, Evanston, IL. 2014c).

133. Scotese, C.R. Atlas of Jurassic paleogeographic maps, PALEOMAP Atlas for ArcGIS, Vol. 4, The Jurassic and Triassic, Maps 32–42, Mollweide projection, (PALEOMAP Project, Evanston, IL. 2014d).

134. Scotese, C.R. Atlas of Permo-Carboniferous Paleogeographic Maps (Mollweide projection), Maps 53–64, Volumes 4, The Late Paleozoic, PALEOMAP Atlas for ArcGIS, (PALEOMAP Project, Evanston, IL. 2014e).
