# Supplementary material for: Fossils reshape the Sternorrhyncha evolutionary tree (Insecta, Hemiptera)
Source: Sci Rep. 2020 Jul 9;10:11390. doi: 10.1038/s41598-020-68220-x (PMC7347605; doi:10.1038/s41598-020-68220-x)
Supplement: Supplementary file 2 — Supplementary Information 2. [file 41598_2020_68220_MOESM2_ESM.pdf]

	Head capsule	Genal cones	Setae on head	Antennal flagellum	Sensory structures on pedicell	Rhinaria on antennae	Antennal processus terminalis	Imaginal compound eyes ommatidia	Imaginal compound eyes dorsoventrally	Imaginal compound eye additional structures	Rostrum placed	Rostrum well developed	Mesonotum	Praescutum	Praescutum	Mesoscutum	Mesoscutellum	Mesopostnotum	Metathoracic tergite	Bases of legs	Fore femora armed with long spines
	**1**	**2**	**3**	**4**	**5**	**6**	**7**	**8**	**9**	**10**	**11**	**12**	**13**	**14**	**15**	**16**	**17**	**18**	**19**	**20**	**21**
Fulgoromorpha	0	0	0	1	1	1	1	0	0	0	0	0	0	-	-	-	-	-	-	0	0
Pincombeomorpha	?	0	?	?	?	?	?	0	0	0	1	0	?	?	?	?	?	?	?	?	0
Coccomorpha	1	0	0	0	0	1	1	1	0	0	1	1	1	0	0	0	0	1	1	1	0
Naibiomorpha	0	0	0	0	0	0	0	0	0	1	1	0	1	0	0	1	0	1	1	1	0
Aphidomorpha	0	0	0/1	0	0	0	0	0	0	1	1	0	1	0	0	1	0	1	0	1	0
Protopsyllidioidea	0	0	0/1	0	0	1	1	0	0	0	0	0	1	0	0	1	0	1	0	0	0/1
Liadopsyllidae	0	0	0	0	0	0	1	0	0	0	2	0	1	1	1	0	0	1	0	0	0
Psylloidea	0	1	0/1	0	0	0	1	0	0	0	2	0	1	1	1	0	0	1	0	0	0
Dinglomorpha	0	0	0	0	0	?	0	0	0	0	1	0	1	1	1	0	1	0	0	0	0
Aleyrodomorpha	0	0	0	0	0	0	0	0	1	0	1	0	1	1	1	0	1	0	0	0	0

	Hind coxae	Tarsi	Basal cell	Vein Pc	Veinlet cua-cup at basal cell	Costal complex of veins	Costal break	Costal cell	ScP	Very basal portion of ScP+R+MP	Stigmal area	MP	Cross vein rp-mp	Areola postica	Clavus	Thickened ambient vein	Wings covered with setae	Hind wings	Halterae	abdomen connection	Hypandrium
	**22**	**23**	**24**	**25**	**26**	**27**	**28**	**29**	**30**	**31**	**32**	**33**	**34**	**35**	**36**	**37**	**38**	**39**	**40**	**41**	**42**
Fulgoromorpha	1	0	0	0	0	1	0	1	0	0	0/1	0	0	0	0	0	0/1	0	0	0	0
Pincombeomorpha	?	0	0	0	0/1	0	0	1	0	0	1	0	0	0	0	0	0	0	0	?	?
Coccomorpha	0	1	1	0	1	0	0	0	0	0	0	1	1	1	1	0	0	1	1	0	0
Naibiomorpha	0	1	1	0	1	0	0	0	0	0	1	1	1	0	1	0	0	1	0	0	0
Aphidomorpha	0	1	1	0	1	0	0	0	0	0	1	0	1	0	1	0	0	1	0	0	0
Protopsyllidioidea	0		0/1	1	0/1	1	0	0	1	0	0	0	0/1	0	0	1	1	0	0	1	1
Liadopsyllidae	0	1	1	1	1	1	0	1	1	0	0	0	1	0	0	1	0	0	0	1	1
Psylloidea	1	1	1	1	1	1	1	1	1	0	0	0	1	0	0	1	1	0	0	1	1
Dinglomorpha	0	1	1	1	1	1	0	0	1	1	0	1	1	1	0	1	0	0	0	0	1
Aleyrodomorpha	0	1	1	1	1	1	0	0	1	0	0	0	1	1	0	1	0	0	0	1	1
